# The uncertainty with using risk prediction models for individual decision making: an exemplar cohort study examining the prediction of cardiovascular disease in English primary care

**DOI:** 10.1186/s12916-019-1368-8

**Published:** 2019-07-17

**Authors:** Alexander Pate, Richard Emsley, Darren M. Ashcroft, Benjamin Brown, Tjeerd van Staa

**Affiliations:** 1 Centre of Health eResearch, School of Health Sciences, Faculty of Biology, Medicine and Health, The University of Manchester, Oxford Road, Manchester, M13 9PL UK; 2 Department of Biostatistics and Health Informatics, Institute of Psychiatry, Psychology and Neuroscience, King’s College London, De Crispigny Park, London, SE5 8AF UK; 3 NIHR Greater Manchester Patient Safety Translational Research Centre, School of Health Sciences, Faculty of Biology, Medicine and Health, The University of Manchester, Oxford Road, Manchester, M13 9PL UK; 4 NIHR School for Primary Care Research, Centre for Primary Care, Division of Population of Health, Health Services Research and Primary Care, Manchester Academic Health Science Centre, University of Manchester, Manchester, M13 9PL UK; 5 Public Health England North West, 3 Piccadilly Place, London Road, Manchester, M1 3BN UK; 6 Division of Pharmacoepidemiology and Clinical Pharmacology, Utrecht Institute of Pharmaceutical Sciences, Utrecht University, Utrecht, Netherlands

**Keywords:** Uncertainty analysis, Cardiovascular disease, Risk prediction, Primary care

## Abstract

**Background:**

Risk prediction models are commonly used in practice to inform decisions on patients’ treatment. Uncertainty around risk scores beyond the confidence interval is rarely explored. We conducted an uncertainty analysis of the QRISK prediction tool to evaluate the robustness of individual risk predictions with varying modelling decisions.

**Methods:**

We derived a cohort of patients eligible for cardiovascular risk prediction from the Clinical Practice Research Datalink (CPRD) with linked hospitalisation and mortality records (*N* = 3,792,474). Risk prediction models were developed using the methods reported for QRISK2 and 3, before adjusting for additional risk factors, a secular trend, geographical variation in risk and the method for imputing missing data when generating a risk score (model A–model F). Ten-year risk scores were compared across the different models alongside model performance metrics.

**Results:**

We found substantial variation in risk on the individual level across the models. The 95 percentile range of risks in model F for patients with risks between 9 and 10% according to model A was 4.4–16.3% and 4.6–15.8% for females and males respectively. Despite this, the models were difficult to distinguish using common performance metrics (Harrell’s *C* ranged from 0.86 to 0.87). The largest contributing factor to variation in risk was adjusting for a secular trend (HR per calendar year, 0.96 [0.95–0.96] and 0.96 [0.96–0.96]). When extrapolating to the UK population, we found that 3.8 million patients may be reclassified as eligible for statin prescription depending on the model used. A key limitation of this study was that we could not assess the variation in risk that may be caused by risk factors missing from the database (such as diet or physical activity).

**Conclusions:**

Risk prediction models that use routinely collected data provide estimates strongly dependent on modelling decisions. Despite this large variability in patient risk, the models appear to perform similarly according to standard performance metrics. Decision-making should be supplemented with clinical judgement and evidence of additional risk factors. The largest source of variability, a secular trend in CVD incidence, can be accounted for and should be explored in more detail.

**Electronic supplementary material:**

The online version of this article (10.1186/s12916-019-1368-8) contains supplementary material, which is available to authorized users.

## Background

Risk prediction models have become an important part of clinical decision-making. They provide a quick and simple way to assess a patient’s risk of a given disease or particular event which can then guide treatment. A recent review by Damen et al. [1] found 363 models for predicting a patient’s risk of developing cardiovascular disease (CVD), and a review by Goldstein et al. found 107 models from 2009 to 2014 that use routinely collected data from electronic health records (EHRs) [2]. In the UK, national guidelines recommend that clinicians use a risk prediction model (QRISK2 [3]) to determine whether to prescribe a statin for primary prevention of CVD (if a patient’s CVD risk is 10% or more [4]). There have also been recent initiatives of promoting public use of similar tools with completing of online questionnaires and provision of individual estimates of ‘Heart Age’ [5, 6]. This has resulted in considerable publicity and concern as four-fifths of those that participated were found to have a heart age which exceeded their chronological age [7, 8], when in reality this is probably not true. The public availability of these algorithms contradicts the NICE guidance, which emphasises the approximate nature of these algorithms when applied to a specific patient and the need for interpreting the risk scores alongside informed clinical judgement [4].

The validity and usefulness of risk prediction models are currently assessed using population-level statistics that measure calibration and discrimination. Calibration [9] is a measure of predictive accuracy assessing whether the average predicted risk is close to the observed risks in the overall population or in subgroups of that population. Discrimination is a relative measure of whether patients with higher risks are more likely to have an event (i.e. in a logistic regression model) or more likely to have an event sooner (i.e. in a survival analysis) than those with lower risks. In logistic regression, the area under the curve [9] can be calculated, whereas for survival models, Harrell’s C is a commonly used metric [10]. One characteristic of note of these measures is that they are population-based and derived from classifying larger groups of patients. They do not provide evidence of the level of uncertainty around a risk prediction for an individual patient beyond the statistical confidence interval. Uncertainty on a patient level may occur if major risk factors are not considered, models are applied outside the setting in which they were developed or different EHR systems or coding dictionaries are being used with varying standards in data collection [11, 12]. Furthermore, modelling decisions such as which variables to include or how to define the cohorts for the development of the models may also yield different risk predictions for the same patient. Variable selection is often based on prior/expert knowledge, which may result in different models depending on which researchers are involved. While data-driven methods exist for variable selection, it is unclear what the best way to do this is and again different methods may result in a different set of predictors. Recent research found that well-established risk prediction models (such as Framingham and QRISK2) provided inconsistent predictions for individuals [13] despite these models having good population-level performance metrics. Uncertainty analyses have been proposed in order to establish whether models can be used for individual decisions [14]. These go beyond the classical statistical confidence interval which evaluates the uncertainty associated with the fitted values, a group mean for all patients with the same covariates. Instead they evaluate the uncertainty associated with other sources such as the modelling decisions that are made.

The objective of this study was to conduct an uncertainty analysis of the QRISK2 risk prediction model for CVD and to evaluate whether modelling decisions, in particular what patient data we choose to include in the model, had a meaningful impact on individual risk predictions (i.e. whether they substantially changed individual risk predictions). We focus in this study on the type of uncertainty which is known as ‘epistemic’ and caused by a lack of knowledge [14], as opposed to aleatory uncertainty, which is inherent due to the complex processes going on in the human body. This study consisted of a comparison of alternative models, evaluating whether they changed individual risk predictions and population-level performance metrics. Clinicians could face substantial uncertainty if alternative models that perform equally well give different predictions for their patients.

## Methods

### Overview of the development of QRISK risk prediction models

The models developed in this paper are based on the QRISK series of models. These CVD risk prediction models were built using routinely collected EHRs from primary care practices in the UK. The second version QRISK2 [3] is currently being used by general practitioners (GPs) in routine clinical practice. QRISK3 [15] was developed in 2017 and is next in line to be used in practice. All individuals aged 25–84 with no medical history of CVD or prior statin treatment are eligible for risk prediction using this model. We have chosen to base the current analysis around these because they are widely used in clinical practice and have been developed and validated in very large populations (QRISK3 was developed in 4,019,956/3,869,847 females and males) reporting strong performance [3, 15, 16]. Variables proposed for inclusion in these models are those that are known or thought to affect cardiovascular disease from literature and NICE guidelines.

### Study population

This study used data from the Clinical Practice Research Datalink [17] (CPRD) linked with Hospital Episode Statistics [18] (HES), mortality records from the Office for National Statistics [19] (ONS) and Townsend deprivation data. CPRD is a primary care database that is representative of the UK in terms of age, sex and ethnicity [17]. It contains the anonymised EHRs from a large group of general practices and is comparable to The Health Improvement Network (THIN) database which was used in the external validation of QRISK2 [20]. The study population was derived using the same definitions as specified in QRISK3 [15], the most recent version. Overall implementation could be followed closely, although code lists for predictor variables and algorithms for deriving test data were not available, therefore differences will exist here. It included patients aged 25–84 with no history of CVD or statin medication prior to the index date. The index date was the latest date of 25th birthday, 1 year of follow-up for a permanently registered patient or 1 Jan. 1998 (study start date). Follow-up ended on the earliest date of the patient’s transfer out of the practice or death, last data collection for practice or study end date of 31 Dec. 2015. The outcome of interest was defined as the time until the first CVD event (transient ischaemic attack, ischaemic stroke or coronary heart disease) identified either through CPRD, HES or ONS records (code lists provided in Additional file 1).

### Definition of different risk prediction models

A series of different risk prediction models were developed in the study population with increasing amounts of information. Each model contained all the same covariates as the previous one, with some extra variables added to the model. Variables beyond those included in QRISK2 or 3 were identified in literature as thought to be predictive, similar to the method for identifying variables for inclusion in QRISK. We emphasise the point that by selecting variables in such a fashion, we are not trying to answer the question ‘what is the best variables to predict CVD with?’, we are asking ‘how sensitive are individual risks to the addition of new variables?’. The following models were fitted:

(i) Model A (same covariates as QRISK2 [3]) including age, body mass index (BMI), atrial fibrillation, cholesterol/high-density lipoprotein (HDL) ratio, chronic kidney disease (CKD, stage 4/5), ethnicity, family history of CVD, treated hypertension, rheumatoid arthritis, systolic blood pressure (SBP), smoking status, type 1 diabetes, type 2 diabetes, Townsend deprivation score
(ii) Model B (same covariates as QRISK3 [15]), covariates added: atypical antipsychotic use, corticosteroid use, CKD (stage 3/4/5 instead of 4/5), erectile dysfunction, HIV/AIDS, migraine, severe mental illness, SBP variability, systemic lupus erythematosus
(iii) Model C included covariates believed to be predictive of CVD risk as identified from literature, covariates added: alcohol abuse [4], anxiety [21], left ventricular hypertrophy [13], number of days with a medical record in year prior to index date [13] and number of prescription items in 1 year prior to index date [13]
(iv) Model D added the calendar time at the patient’s index date to account for a secular trend in CVD [22]
(v) Model E added the region the patient resides in to account for regional variation in CVD incidence [23] (taken at the strategic health authority (SHA) level); after a restructuring in 2013, SHAs now represent 10 purely geographical locations across England [24]

The same methods were used to derive variables as in QRISK3 when possible. Detailed information on the derivation of all covariates can be found in Additional file 1.

### Development of risk prediction models

We used multiple imputation by chained equations to impute missing data for BMI, SBP and SBP variability, cholesterol, HDL, smoking status and ethnicity. All predictor variables from model E were included as predictors in the imputation procedure, as well as the Nelson Aalen estimate of the cumulative baseline hazard at the point of censoring or an event. The program used to impute the data was the R package MICE [25]. We imputed 20 datasets and carried out 20 iterations for each dataset. Full details about the imputation process can be found in Additional file 2. The same randomly selected 200,000 patients were removed from each dataset, with the remaining patients making up the development cohort. All models were developed on the same set of 20 imputed datasets. For model development, Cox proportional hazards models were fitted, similar to QRISK, predicting the 10-year risks of developing CVD and estimating the hazard ratios (HRs) for each of the covariates. Models were developed separately for females and males. For model E, a random intercept model was fitted for region (strategic health authority level). Fractional polynomials for age and BMI were tested when developing model A using the R package mfp [26], and these fractional polynomials were used in all subsequent models. Fractional polynomials were tested for a secular trend in model D and were used in all subsequent models. When developing risk scores, survival estimates were combined using Rubin’s rules on the log(−log) scale [27].

### Validation of models

Key aspects of data and model B were compared with QRISK3 to highlight that the cohort used to develop the models was similar. We have chosen to make these comparisons with QRISK3 as the cohort is defined over the same time period. We compared incidence rates, distribution of covariates, HRs and predicted risks. This was done for model B, as this was developed using the same covariates as QRISK3, the comparator. The calibration of model B was also tested using internal validation with 200,000 randomly sampled patients to make up the test data and the remaining patients to develop the models (split sample approach). Average predicted risks were compared with the Kaplan-Meier survival estimate at 10 years to assess calibration across groups defined by the 10th percentile of risk.

Various model performance measures evaluated the performance of all our models [28–30]. These included a variety of discrimination measures (Harrell’s *C*_H_ [10], Uno’s *C*_U_ [31], Gonen and Heller’s *C*_GH_ [32] and Royston and Saurbrei’s *D* measure [33]), two measures of explained randomness (Kent and O’Quigley’s *ρ*_w,a_ [34], O’Quigley et al.’s *ρ*_*k*_ [35]), one measure of predictive accuracy (Integrated Brier Score (IBS) [36, 37]), and four measures of explained variation (Kent and O’Quigley’s *R*^2^_PM_ [38], then Roystons *R*^2^, Roystons *R*^2^_*D*_ [39] and *R*^2^_IBS_ [36], which are based on the measures *ρ*_*k*_, *D* and IBS respectively). These were calculated to validate the models, but also as a key outcome in our study. We were interested in knowing to what extent the model performance metrics change between models if those models are predicting sizably different risks for individuals. While these metrics are not designed to assess model performance on an individual level, they are commonly used to evaluate models which are in turn used for individualised risk prediction. It is therefore important to know how sensitive they are to changes in risk on that individual level. We therefore report a range of metrics to help highlight which types of metric may best explain these changes in individual risk. When possible, performance metrics were calculated using a split sample approach (validation cohort size 200,000). *ρ*_*k*_, *R*^2^_*K*_ and *C*_GH_ are based on model features rather than event and censoring times, and therefore, the split sample approach does not apply. *C*_GH_ was calculated on the model developed on a sample of 200,000 patients as the algorithm used was unable to handle larger sample sizes.

The three concordance indexes estimate the probability that for a randomly selected pair of patients, the higher risk patient will have the event sooner. The range of values is 0.5–1, with a higher value indicating better performance. The *D* statistic, which calculates the log HR between two groups of patients split at the median of the linear predictor, does not have this restriction and may take values between 0 and infinity. Austin et al. found that *C*_H_ and *C*_U_ were equally sensitive to the inclusion of new novel risk factors and were more sensitive than *C*_GH_. They also echo the sentiments of Harrell and Uno that concordance statistics may not be sensitive when choosing between competing models and measures of explained variation may be more sensitive in detecting differences in predictive ability. The measures of explained variation and explained randomness may take values between 0 and 1. Choodari-Oskooei et al. [28] recommended using explained variation measures *R*^2^_PM_ and *R*^2^_*D*_ for best meeting their criteria (independence from censoring, monotonicity, interpretability and robustness against outliers). For explained randomness *ρ*_*w*,*a*_ is recommended by both Choodari-Oskooei et al. [40] and Austin et al. [30], despite their differing criteria of importance. This measure is very similar to *R*^2^_PM_, where the variance error term *σ*^2^/6 is replaced by 1. Finally, the integrated brier score is included as it has a different aim, which is to calculate the probability of correctly predicting an event. The development and validation of models was checked against the recommendations for reporting in the TRIPOD statement (Additional file 3).

### Comparison of predicted risks between different models

After developing the models, the next step was to produce risk scores, replicating the process of someone having their risk assessed in practice. In this situation, if a patient has missing data for specific covariates, the QRISK calculator will impute this using mean imputation based on age, sex and ethnicity [41]. This involved setting all originally missing values of BMI, cholesterol HDL ratio, SBP and SBP variability, back to missing, and then imputing these using mean imputation based on age, sex and ethnicity, giving one mean imputed dataset. The same 200,000 patients were then extracted from the mean imputed dataset giving the test cohort. For each patient in the test cohort, a predicted risk according to each of model A–E was then generated. This is like a split sample approach, apart from the fact that the imputation method for the development cohort and test cohort is different (as is the case in practice).

Finally, risks were also generated using model E, but for a test cohort made up of 200,000 patients from one of the multiply imputed datasets, rather than mean imputed. This represents a best estimate of the true values of each patient’s missing data. The aim of this was to understand how much variation in patient risk may be masked by using mean imputation to generate a risk, as opposed to prospectively collecting their real values, as recommended by NICE. This will be referred to as model F.

The predicted CVD risks for each patient were compared between model A and models B–F. We started with model A as this model replicates the risk scores developed using QRISK2, which is the model currently used in practice. This evaluated the magnitude in which risks for an individual patient change dependent on what patient characteristics were introduced into the model. Patients were grouped into risk groups of width 1% according to their risk in model A. Then for models B–F, we provide histograms to illustrate the distribution of risks for patients from the same group, report the 2.5–97.5 percentile range for each group (average 95% CI according to model A also provided for comparison) and report the proportion of patients from each group with a risk above or below 10%, which is the threshold for being eligible for a statin prescription in England [4].

The final analysis consisted of the extrapolation of results to the population of England in order to assess what proportion of patients would have their treatment pathway altered depending on the model used. We extrapolated the proportion of patients eligible for CVD risk prediction in CPRD on 12 Jan. 2016 to the population in England [42] and then estimated the level of reclassification when using model F instead of model A (QRISK2). Eligibility for patients on 1 Jan. 2016 was the same as in the development cohorts, except the index date was set to 1 Jan. 2016 for all patients. This dataset was mean imputed when calculating risks according to model A–E, and one stochastically imputed dataset when calculating risks according to model F.

### Sensitivity analyses

We found a large effect of a secular trend in CVD incidence, resulting in 56% of the patients from the 2016 cohort to be reclassified from above to below the statin treatment threshold of 10% (see results—extrapolation to English population). We therefore ran two sensitivity analyses to validate this finding. First, we verified the existence of the secular trend reporting crude incidence rates per calendar year amongst the model derivation cohort. For the second, we evaluated the existence of the secular trend in a cohort of statin users. For this cohort, all patients that were eligible for linkage and had more than one statin prescription between ages 25 and 85 and dates 1 Jan. 1998 and 31 Dec. 2015 were included. Follow-up started on the first statin prescription date and ended after a 6-month gap with no prescription. A patient could re-enter the cohort if they initiated statins again. A patient was not followed up after the event of interest (CVD). We check for the presence of this trend amongst the statin users’ cohort as the secular trend in CVD incidence could be explained by an increase in statin use.

To analyse this data, each patient’s follow-up was segmented into time followed up in each calendar year. It was also recorded whether a patient had an incident CVD event in that calendar year. We then fit a Poisson model to the data, outcome being the CVD event, adjusting for calendar year and using the time at risk in each year as an offset. This was done for the development cohort and the statin users’ cohort. Another model was also fit to the statin users’ cohort adjusting for the risk score at the start of the period of statin treatment as well. This model attempts to find out whether the secular trend could also be explained by better prescribing of statins to those who are at high risk, through the use of models such as QRISK. The secular trend would only be of interest if it is still present in this model, which accounts for a potential change in the use of statins.

### Software

Extraction of data and cohort derivation was done using SAS/STAT software, version 9·4 for Windows. SAS and all other SAS Institute Inc. product or service names are registered trademarks or trademarks of SAS Institute Inc., Cary, NC, USA. All analyses were conducted using R version 3.4.2.

## Results

### Validation of the models

CPRD contained 6,869,457 patients with > 1 day follow-up aged 25–84 during the study period. Of these, 3,855,660 (from 392 practices) were eligible for linkage to HES, ONS and Townsend quintiles and were without history of CVD or statin treatment at baseline. Table 1 contains the baseline characteristics for all patients who met the study eligibility criteria, which includes all patients which we generate risk scores for. There was 42.07% and 38.21% of data recorded for ethnicity for the male and female cohorts respectively, 68.83% and 53.62% for BMI, 38.48% and 35.71% for cholesterol/HDL ratio, 81.01% and 59.21% for SBP, 50.39% and 20.94% for SBP variability and 75.18% and 65.17% for smoking status. The mean ages were 43.07 and 41.81 for females and males and the mean BMI was 25.60 and 26.12, while cholesterol/HDL ratio was 3.72 and 4.48 respectively. More importantly, we found these values to match closely with those from the derivation cohort of QRISK3 (age 43.3 and 42.6, BMI 25.4 and 25.6, cholesterol/HDL ratio 3.7 and 4.4 respectively), a full comparison given in Additional file 4: Table S1. The prevalence of medical history variables was broadly similar with that in QRISK3. Similarly, the incidence rate of CVD matched closely for both datasets (for females, there were 6.19 CVD cases per 1000 person-years in our study population compared to 6.27 in QRISK3; for males, these were 8.18 vs 8.24 respectively), shown in Additional file 4: Tables S2.1 and S2.2.

**Table 1:** CVD incidence and baseline characteristics of the entire study population

	CPRD female *N* = 1,965,078	CPRD male *N* = 1,890,582
Outcome variables		
Incident CVD cases	86,547	107,051
Person years	13,801,919	12,977,235
Rate per 1000 person-years	6.27	8.24
Demographics		
Age	43.07 (15.94)	41.84 (14.57)
Ethnicity		
Recorded	42.07%	38.21%
White/not recorded	94.12%	94.48%
Indian	1.14%	1.19%
Pakistani	0.45%	0.49%
Bangladeshi	0.14%	0.19%
Other Asian	0.84%	0.78%
Black	1.73%	1.52%
Chinese	0.33%	0.23%
Other	1.27%	1.12%
Test data		
BMI	25.60 (5.60)	26.12 (4.54)
Cholesterol/HDL ratio	3.72 (1.20)	4.48 (1.40)
SBP	123.91 (18.28)	130.03 (16.48)
SBP variability	9.47 (5.98)	10.13 (6.80)
Smoking status	Never = 56.04%, Ex = 16.97%, Current = 26.99%	Never = 46.63%, Ex = 17.48%, Current = 35.99%
Medical history		
Atrial fibrillation	0.44%	0.57%
Atypical antipsychotic medication use	0.30%	0.33%
Chronic kidney disease		
Stage 3/4/5	0.45%	0.32%
Stage 4/5	0.12%	0.15%
Corticosteroid use	0.48%	0.30%
Erectile dysfunction	NA	1.45%
Family history of CVD	15.08%	11.02%
HIV/AIDS	0.06%	0.09%
Migraine	7.27%	2.94%
Rheumatoid arthritis	0.69%	0.26%
Severe mental illness	8.63%	4.59%
Systemic lupus erythematosus	0.10%	0.01%
Treated hypertension	6.18%	4.50%
Type 1 diabetes	0.21%	0.28%
Type 2 diabetes	1.16%	1.42%
Variables not in QRISK		
Number medical records in the previous year	14.94 (13.97)	8.83 (11.45)
> 50 medical records in the previous year	2.84%	1.37%
Number of prescription items in the previous year	9.60 (19.87)	5.72 (16.00)
Number with > 50 prescription items in the previous year	3.49%	2.04%
Alcohol abuse	0.65%	1.46%
Anxiety	13.44%	7.96%
Left ventricular hypertrophy	0.14%	0.18%
Region		
North East	1.89%	1.96%
North west	13.10%	13.38%
Yorkshire and the Humber	3.93%	3.85%
East Midlands	3.14%	3.23%
West Midlands	11.04%	11.28%
East of England	11.67%	11.68%
South west	11.99%	11.88%
South Central	12.84%	12.81%
London	17.52%	17.18%
South East Coast	12.88%	12.74%

The HRs for model B (Additional file 4: Table S3) were generally consistent with those reported in QRISK3. The HRs for covariates introduced for models C, D and E are reported in Table 2. All introduced covariates had a sizeable effect on risk. For example, the HRs for patients in the North West were 1.17 for females and 1.14 for males, compared to 0.92 and 0.94 respectively for patients from South Central. The HR associated with calendar time was also large, with a 0.95 and 0.96 reduction for females and males respectively each year.

**Table 2:** HRs (95% CI) of fixed and random effects introduced into models C, D and E. HRs reported are all from model E

	Female	Male
Fixed effects		
Alcohol abuse	1.36 (1.25–1.48)	1.32 (1.25–1.39)
Anxiety	1.10 (1.08–1.13)	1.10 (1.07–1.12)
Left ventricular hypertrophy	1.65 (1.53–1.78)	1.67 (1.56–1.80)
> 50 medical records in the year prior to the index date	1.30 (1.25–1.36)	1.25 (1.18–1.31)
> 50 prescription items in the year prior to the index date	1.55 (1.51–1.59)	1.49 (1.44–1.54)
Calendar time (by year)	0.96 (0.95–0.96)	0.96 (0.96–0.96)
Region (random effect)		
North East	1.07 (1.00–1.14)	1.09 (1.08–1.09)
North west	1.17 (1.11–1.24)	1.14 (1.13–1.15)
Yorkshire and the Humber	1.11 (1.05–1.19)	1.09 (1.08–1.10)
East Midlands	1.00 (0.93–1.06)	0.99 (0.98–0.99)
West Midlands	0.99 (0.94–1.05)	0.99 (0.99–1.00)
East of England	0.94 (0.89 1.00)	0.94 (0.93–0.94)
South west	0.98 (0.92 1.04)	0.99 (0.99–0.99)
South Central	0.92 (0.87–0.98)	0.94 (0.94–0.95)
London	0.89 (0.84–0.95)	0.88 (0.88–0.89)
South East Coast	0.96 (0.90–1.02)	0.97 (0.97–0.97)

**Fig. 1:**
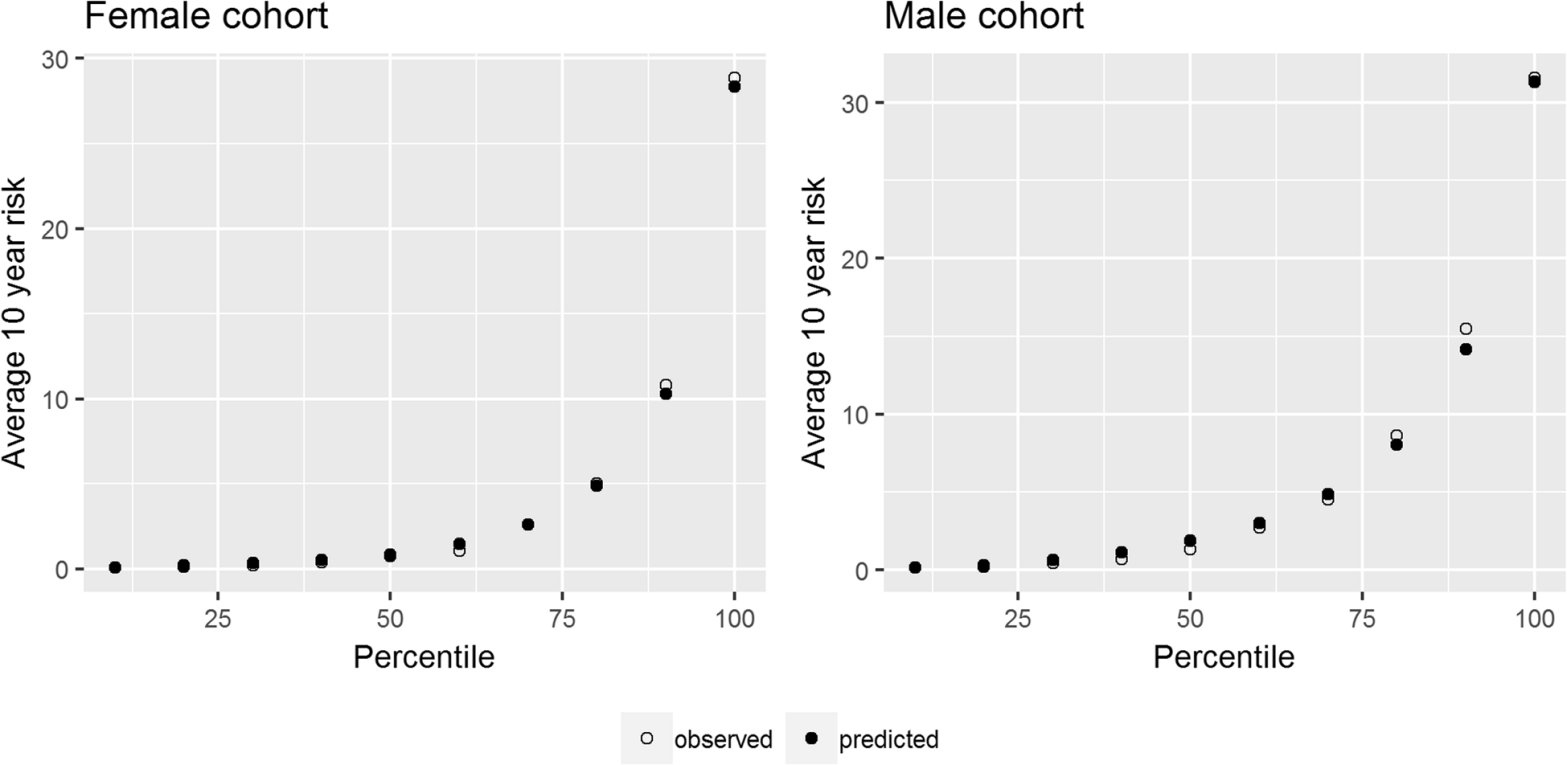
Calibration plots by 10th percentile of risk for model B

The calibration plots for model B showed overall good calibration (Fig. 1), which is expected considering these are optimistic calibration plots (internal validation only). The female model is very well calibrated with the calibration error no larger than 0.5% for any 10th percentile group. The largest miscalibration for the male model is for group 9, an under prediction by 1.29%.

The overall performance metrics calculated for each of the models are given in Table 3. The largest increase is in *D* and *R*^2^_*D*_ (which is derived from *D*), which increase from 2.39 to 2.55 and 0.58 to 0.61 (females) across the models respectively. There was little change in any of the three *C* statistics across the different models. While Uno’s *C*, *C*_U_, went from 0.85 to 0.88 for the female cohort, there was not a consistent upward trend in the male models. Harrell’s *C*, the most commonly reported metric, was very insensitive to the model choice. Measures of explained variation and randomness showed an upward trend from model A to model F, while measures derived from the IBS were not sensitive to model choice.

**Table 3:** Performance metrics for each of the models

Measure	Model A	Model B	Model C	Model D	Model E	Model F
Female						
IBS	0.02	0.02	0.02	0.02	NA	NA
*R*^2^_IBS_	0.12	0.13	0.13	0.13	NA	NA
*R*^2^_PM_	0.65	0.65	0.66	0.67	0.67	0.67
*Ρ*^2^_*k*_	0.85	0.86	0.86	0.86	0.86	NA
*Ρ*_*w*,*a*_	0.76	0.76	0.76	0.77	0.77	0.77
*R*^2^	0.62	0.62	0.63	0.63	0.64	NA
*D*	2.39	2.42	2.49	2.52	2.52	2.55
*R*^2^_D_	0.58	0.58	0.60	0.60	0.60	0.61
*C*_H_	0.86	0.87	0.87	0.87	0.87	0.87
*C*_U_	0.85	0.86	0.86	0.86	0.86	0.88
*C*_GH_	0.81	0.82	0.82	0.82	NA	NA
Male						
IBS	0.03	0.03	0.03	0.03	NA	NA
*R*^2^_IBS_	0.12	0.12	0.12	0.12	NA	NA
*R*^2^_PM_	0.62	0.63	0.63	0.63	0.64	0.64
*Ρ*^2^_*k*_	0.78	0.79	0.79	0.79	NA	NA
*Ρ*_*w*,*a*_	0.73	0.73	0.73	0.74	0.74	0.75
*R*^2^	0.49	0.49	0.50	0.50	NA	NA
*D*	2.12	2.12	2.18	2.21	2.21	2.24
*R*^2^_*D*_	0.52	0.52	0.53	0.54	0.54	0.55
*C*_H_	0.84	0.84	0.84	0.84	0.84	0.85
*C*_U_	0.75	0.74	0.74	0.74	0.74	0.77
*C*_GH_	0.81	0.81	0.81	0.82	NA	NA

### Analysis of risk scores

**Fig. 2:**
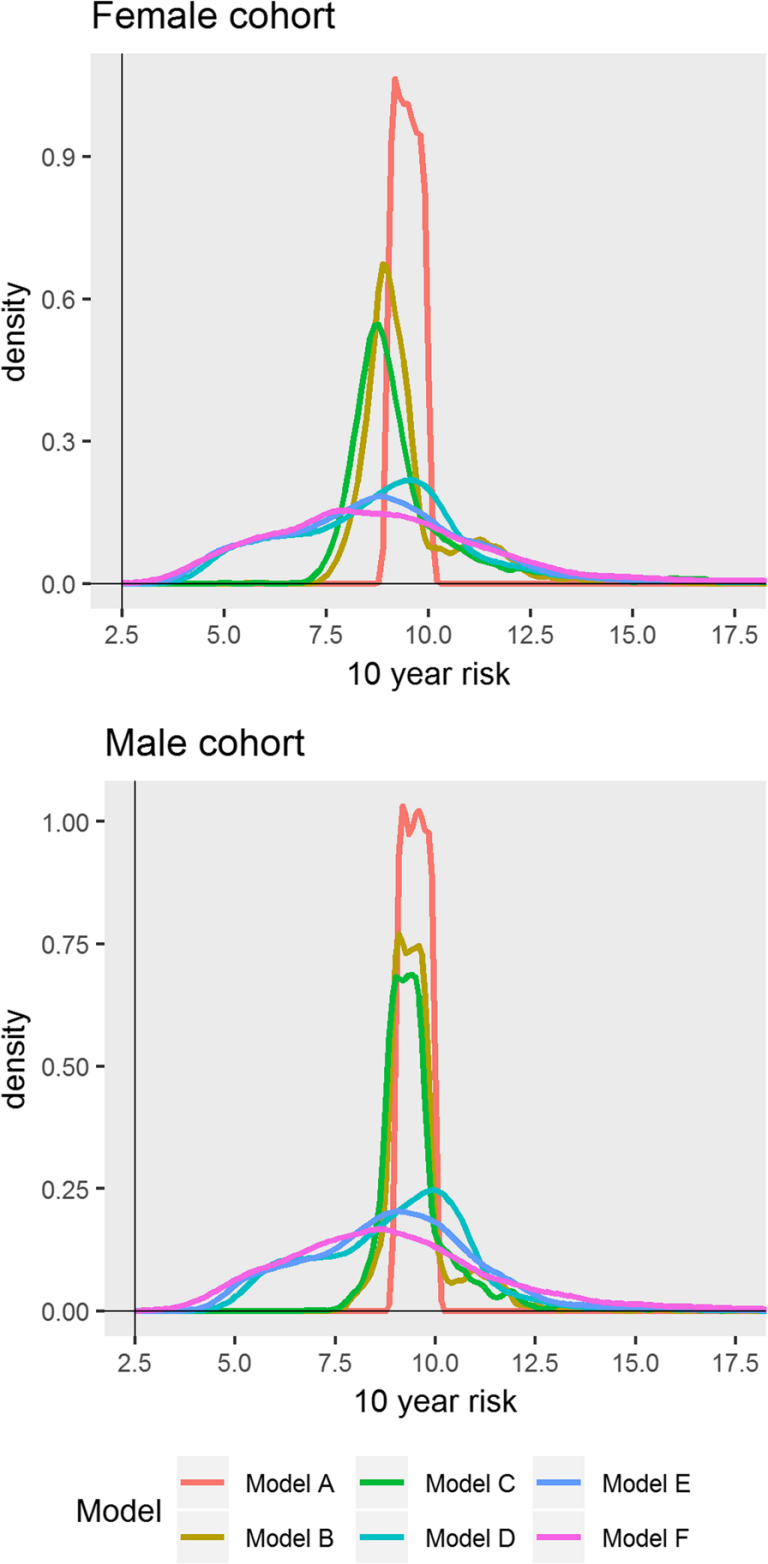
Distribution of risks according to each model for those with risk 9–10% in model A

Table 4 shows the distribution of changes in predicted CVD risks when using models B–F instead of model A. Females with a risk between 9 and 10% with model A (QRISK2) were found to have risks with a 95% percentile range of 8.0 to 13.6 with model B (QRISK3) and range of 4.4 to 16.5% with model F. The impact of the choice of model on the distribution of risks increased with higher CVD risks. For females with a risk of 19 to 20% with model A, their risks were between 9.6 and 34.6 (95% percentile) when using model F. These are shown graphically in Fig. 2.

**Table 4:** Distribution of risks (2.5th and 97.5th percentile) of patients in the test cohort according to each model, stratified by their risk in model A, and average 95% CI for risks in model A

	Female cohort						Male cohort					
10-year risk using model A	Average 95% CI in model A	Percentile range model B	Percentile range model C	Percentile range model D	Percentile range model E	Percentile range model F	Average 95% CI in model A	Percentile range model B	Percentile range model C	Percentile range model D	Percentile range model E	Percentile range model F
0–1%	0.3–0.4%	0.1–0.9%	0.1–0.9%	0.1–1.0%	0.1–1.0%	0.1–1.0%	0.3–0.4%	0.1–1.0%	0.1–1.0%	0.0–1.0%	0.0–1.0%	0.0–1.1%
1–2%	1.4–1.5%	1.0–2.2%	1.0–2.3%	0.6–2.3%	0.6–2.4%	0.6–2.6%	1.4–1.6%	1.0–2.0%	1.0–2.1%	0.7–2.2%	0.7–2.2%	0.6–2.7%
2–3%	2.3–2.6%	1.9–3.6%	1.9–3.9%	1.2–3.9%	1.1–4.0%	1.1–4.4%	2.4–2.6%	2.0–3.2%	2.0–3.3%	1.3–3.4%	1.2–3.5%	1.1–4.4%
3–4%	3.3–3.6%	2.8–5.0%	2.7–5.5%	1.7–5.4%	1.6–5.6%	1.5–6.1%	3.3–3.6%	2.9–4.5%	2.9–4.8%	1.9–4.7%	1.8–5.0%	1.6–6.0%
4–5%	4.3–4.7%	3.7–6.5%	3.6–7.4%	2.3–7.2%	2.2–7.3%	2.1–8.0%	4.3–4.7%	3.9–5.7%	3.8–6.2%	2.5–6.0%	2.4–6.3%	2.1–7.5%
5–6%	5.3–5.7%	4.5–7.8%	4.4–8.9%	2.8–8.5%	2.6–8.8%	2.5–9.5%	5.3–5.7%	4.8–6.9%	4.7–7.7%	3.1–7.6%	2.9–7.6%	2.5–9.3%
6–7%	6.2–6.8%	5.4–9.3%	5.3–10.6%	3.3–10.0%	3.1–10.1%	3.0–11.3%	6.2–6.7%	5.7–8.2%	5.6–8.9%	3.7–8.9%	3.5–9.0%	3.1–11.1%
7–8%	7.2–7.8%	6.3–10.4%	6.1–11.8%	3.8–11.5%	3.6–11.7%	3.5–12.6%	7.2–7.8%	6.6–9.5%	6.5–10.7%	4.3–10.4%	4.1–10.6%	3.7–12.9%
8–9%	8.2–8.8%	7.1–11.7%	6.8–14.3%	4.3–13.3%	4.1–13.3%	4.0–14.6%	8.2–8.8%	7.5–10.6%	7.5–11.8%	4.9–11.5%	4.6–11.9%	4.2–14.1%
9–10%	9.1–9.9%	8.0–13.5%	7.7–16.1%	4.9–15.0%	4.6–15.5%	4.4–16.3%	9.2–9.8%	8.4–11.8%	8.3–13.8%	5.5–13.2%	5.1–13.7%	4.6–15.8%
10–11%	10.1–10.9%	8.8–14.5%	8.5–16.8%	5.3–16.6%	5.1–16.8%	4.9–18.1%	10.1–10.8%	9.2–13.0%	9.0–15.3%	6.0–14.9%	5.6–15.1%	5.2–17.8%
11–12%	11.1–11.9%	9.8–16.3%	9.4–19.6%	5.9–19.3%	5.6–20.1%	5.4–21.1%	11.1–11.9%	10.1–14.2%	9.9–16.5%	6.7–15.8%	6.3–16.0%	5.9–18.9%
12–13%	12.1–12.9%	10.7–17.9%	10.1–21.3%	6.4–20.5%	6.0–21.5%	5.8–22.5%	12.1–12.9%	11.0–15.5%	10.8–17.8%	7.3–17.0%	6.8–17.8%	6.4–20.5%
13–14%	13.0–14.0%	11.4–18.5%	10.9–21.5%	7.1–21.1%	6.7–22.6%	6.6–23.3%	13.1–14.0%	12.0–16.8%	11.8–19.6%	8.0–19.2%	7.5–19.9%	6.9–22.6%
14–15%	14.0–15.0%	12.2–19.7%	11.6–23.3%	7.5–22.5%	7.3–22.7%	6.9–24.3%	14.0–15.0%	12.8–17.8%	12.6–21.7%	8.5–20.3%	8.0–20.8%	7.5–23.3%
15–16%	15.0–16.0%	13.1–22.0%	12.3–26.5%	8.2–26.3%	7.7–27.2%	7.6–28.1%	15.0–16.0%	13.5–19.2%	13.3–22.8%	9.0–21.7%	8.6–22.2%	8.1–23.8%
16–17%	15.9–17.1%	13.9–22.1%	13.0–27.4%	8.5–26.9%	8.0–27.5%	7.9–28.1%	16.0–17.0%	14.6–20.4%	14.2–24.0%	9.8–24.0%	9.2–24.7%	8.7–27.4%
17–18%	16.9–18.1%	14.9–23.9%	14.1–27.9%	9.2–28.9%	8.8–28.7%	8.7–29.5%	16.9–18.1%	15.3–22.8%	15.0–26.9%	10.3–25.5%	9.7–25.5%	9.2–28.6%
18–19%	17.9–19.2%	15.7–25.4%	14.8–30.0%	9.8–29.8%	9.6–29.7%	9.1–32.7%	17.9–19.1%	16.3–23.8%	15.8–26.7%	10.9–26.8%	10.4–26.7%	10.0–29.5%
19–20%	18.8–20.2%	16.6–25.8%	15.7–32.1%	10.5–32.6%	10.0–33.8%	9.7–34.4%	18.9–20.2%	17.2–25.2%	16.7–28.7%	11.5–29.5%	10.9–28.9%	10.4–32.1%

Table 5 summarises the number of patients in the study population who were reclassified with models B–F based on a treatment threshold of 10%. In the female cohort, 8% of those with a CVD risk between 7 and 8% with model A were reclassified to a risk of ≥ 10% with model F (for risks between 8–9% and 9–10%, this was 17% and 28% respectively). Substantially more patients were reclassified downward with predicted risks reduced. In the female cohort, 32% of those with a risk between 12 and 13% were reclassified to a risk of < 10% with model F (for risks between 11–12% and 10–11%, this was 43% and 57% respectively). Similar effects on the risk scores were found amongst the male cohort.

**Table 5:** Numbers and percentages of patients who cross the treatment threshold (10-year CVD risk of 10%) when using models B–F instead of model A

	Predicted 10-year CVD risk according to model A (QRISK2)						Predicted 10-year CVD risk according to model A (QRISK2)					
	5–6%	6–7%	7–8%	8–9%	9–10%	< 10%	10–11%	11–12%	12–13%	13–14%	14–15%	> 10%
Female (*N* = 200,000)												
*N*	5163	4356	3561	3080	2602	169,491	2321	2086	1849	1617	1525	30,509
Model B	21 (0%)	64 (1%)	105 (3%)	392 (13%)	547 (21%)	1140 (1%)	1000 (43%)	114 (5%)	3 (0%)	2 (0%)	4 (0%)	1128 (4%)
Model C	73 (1%)	142 (3%)	220 (6%)	374 (12%)	592 (23%)	1446 (1%)	1260 (54%)	264 (13%)	33 (2%)	7 (0%)	5 (0%)	1575 (5%)
Model D	63 (1%)	108 (2%)	206 (6%)	388 (13%)	689 (26%)	1498 (1%)	1204 (52%)	757 (36%)	461 (25%)	286 (18%)	203 (13%)	3252 (11%)
Model E	69 (1%)	116 (3%)	236 (7%)	466 (15%)	703 (27%)	1649 (1%)	1276 (55%)	833 (40%)	533 (29%)	320 (20%)	223 (15%)	3645 (12%)
Model F	100 (2%)	184 (4%)	310 (9%)	540 (18%)	765 (29%)	1978 (1%)	1328 (57%)	906 (43%)	589 (32%)	367 (23%)	253 (17%)	3953 (13%)
Male (*N* = 200,000)												
*N*	7422	6077	5364	4483	3922	158,036	3355	3108	2710	2421	2283	41,964
Model B	17 (0%)	33 (1%)	91 (2%)	255 (6%)	583 (15%)	983 (1%)	733 (22%)	51 (2%)	0 (0%)	0 (0%)	0 (0%)	784 (2%)
Model C	37 (0%)	86 (1%)	200 (4%)	318 (7%)	728 (19%)	1384 (1%)	1042 (31%)	95 (3%)	4 (0%)	1 (0%)	1 (0%)	1143 (3%)
Model D	25 (0%)	63 (1%)	164 (3%)	341 (8%)	1220 (31%)	1823 (1%)	1625 (48%)	1008 (32%)	627 (23%)	375 (15%)	266 (12%)	4146 (10%)
Model E	30 (0%)	66 (1%)	191 (4%)	505 (11%)	1146 (29%)	1953 (1%)	1792 (53%)	1129 (36%)	705 (26%)	435 (18%)	302 (13%)	4744 (11%)
Model F	126 (2%)	267 (4%)	483 (9%)	750 (17%)	1146 (29%)	2850 (2%)	1896 (57%)	1337 (43%)	888 (33%)	539 (22%)	375 (16%)	5563 (13%)

### Extrapolation to English population

**Fig. 3:**
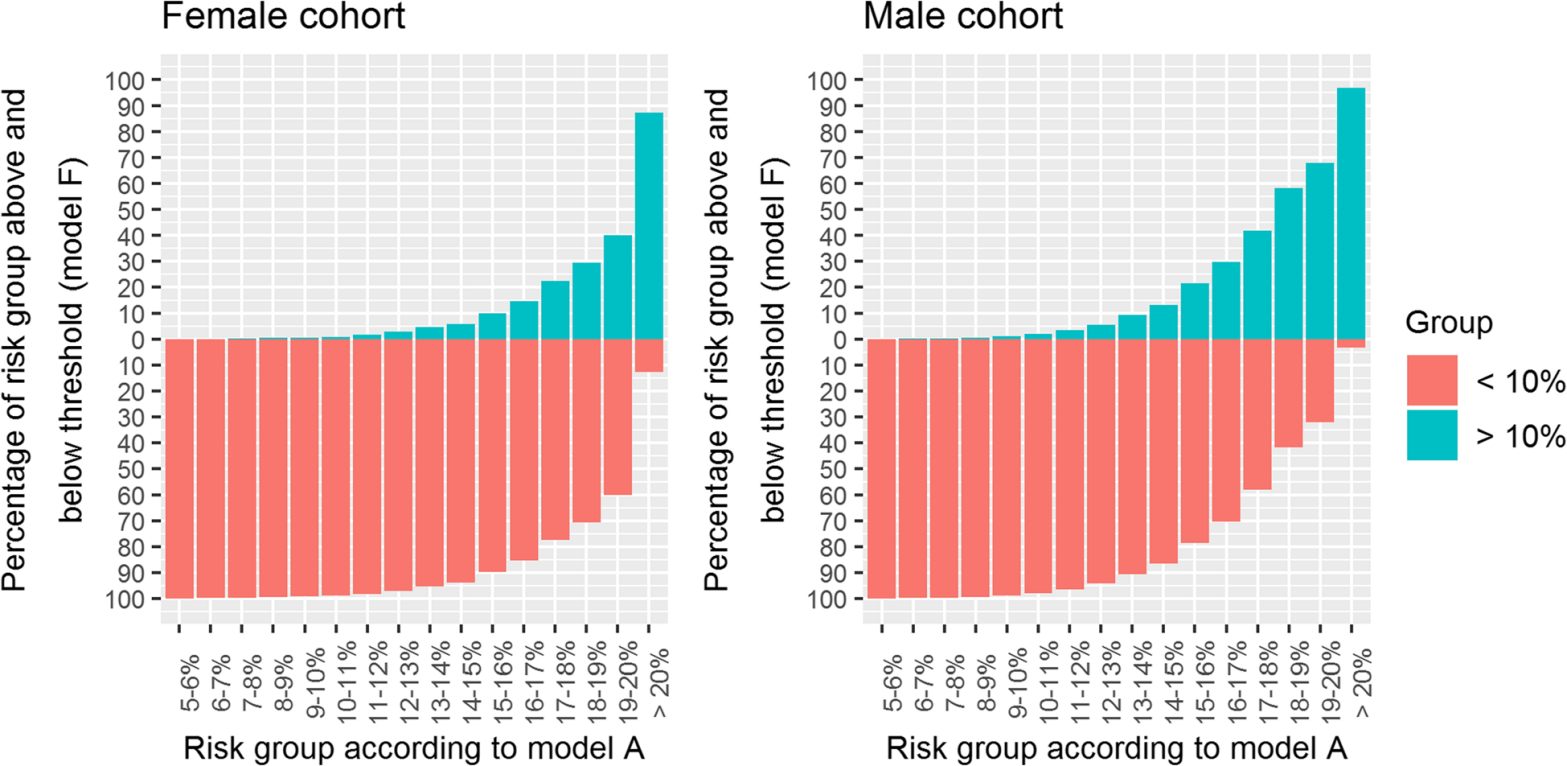
Percentages of patients registered 1 Jan. 2016 who cross the treatment threshold when using model F

Figure 3 shows the proportion of patients reclassified from each risk group when model F is used, applied to the cohort of patients eligible in CPRD for risk assessment on 1 Jan. 2016. When using model F, there was a substantive reclassification downwards across the higher risk categories, in which 64% of females and 52% of males with a risk > 10% would no longer be eligible for statin treatment (Additional file 4: Table S4). This shift is caused by the introduction of the secular trend. When extrapolating results to the population of England, there were 37,273,200 people aged 25–84 in England [42] in 2016 and 29,382,463 would have been eligible for risk assessment using QRISK2 (79% of patients registered on 1 Jan. 2016 were eligible). 6,652,920 of these patients would be classified as high CVD risk (≥ 10%) using model A (QRISK2). If model F was used, 3,792,474 (57%) of them would be reclassified downwards and cross the treatment threshold. The 57% is calculated as the average of the 64% of females and 52% of males, weighted by the female to male ratio. A full breakdown of these calculations and data used to derive Fig. 3 is in Additional file 4: Table S4 (additional text).

### Post hoc analyses of the secular trend

**Fig. 4:**
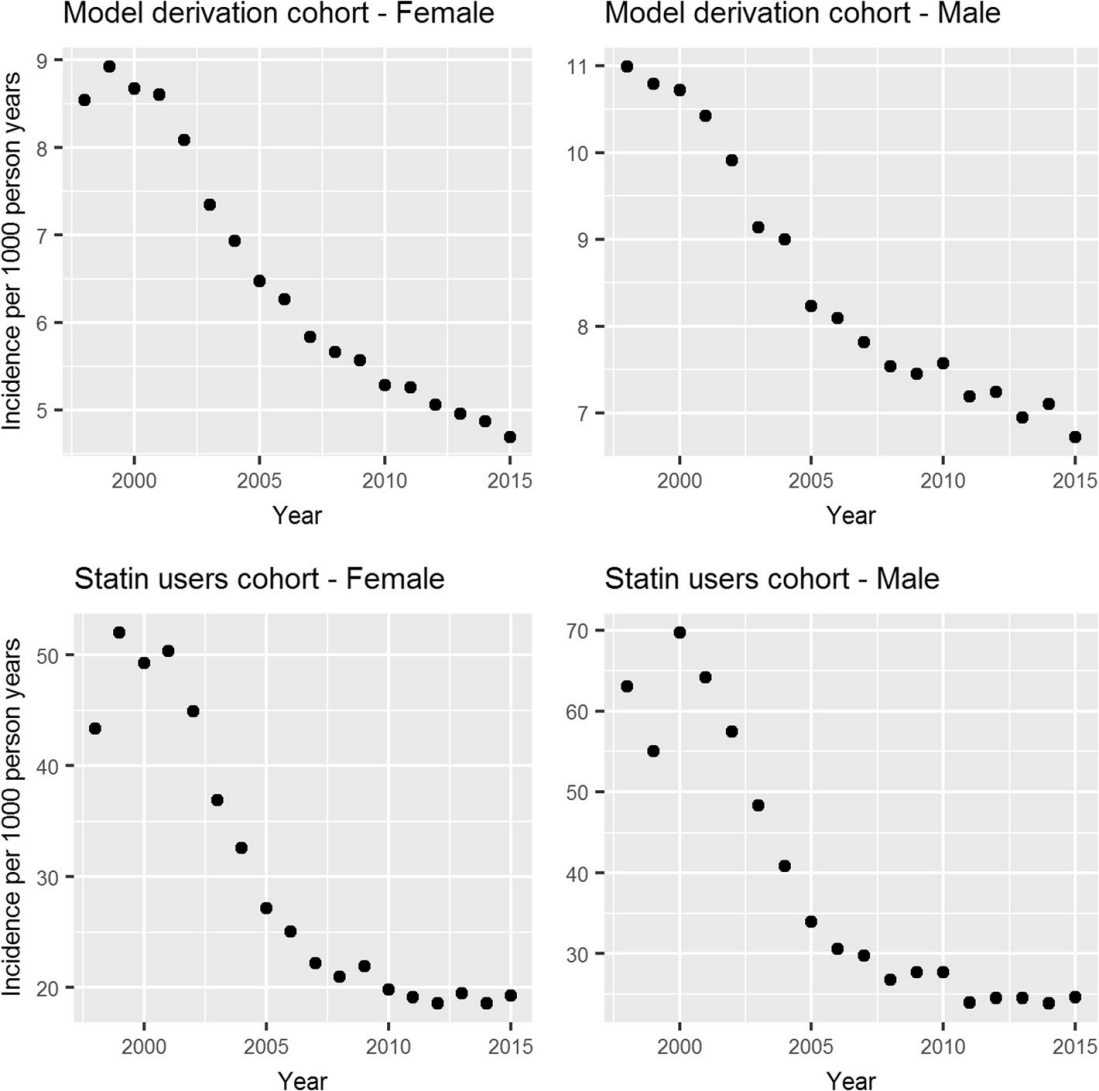
The secular trend in CVD incidence in the model derivation cohort and the statin users’ cohort

There was a strong secular trend in CVD incidence in both the female and male derivation cohorts as can be seen in Fig. 4. The RR was 0.96 (0.96–0.96) and 0.97 (0.97–0.97) annually for females and males respectively (Table 6). A stronger trend was found in the cohort of statin users, with a RR of 0.94 (0.94–0.94) for both cohorts. Adjusting for baseline QRISK2 score, the annual reduction in CVD incidence was unchanged from 0.94 (0.94–0.94) for the female cohort and changed slightly to 0.94 (0.94–0.95) for the male cohort.

**Table 6:** Relative rates (95% CI) associated with the calendar year and risk at start of statin treatment period, in Poisson models modelling CVD incidence

	Female		Male	
Model	Calendar year	Risk at start of treatment	Calendar year	Risk at start of treatment
Development cohort	0.96 (0.96–0.96)	NA	0.97 (0.97–0.97)	NA
Statin users cohort	0.94 (0.93–0.94)	NA	0.94 (0.94–0.94)	NA
Statin users cohort (also adjusting for 10-year risk at inception into cohort)	0.94 (0.94–0.94)	1.02 (1.02–1.03)	0.94 (0.94–0.94)	1.02 (1.02–1.02)

## Discussion

In this study, we assessed the uncertainty in individual risk predictions by using different modelling approaches. A large amount of variability in individual risk predictions was found when taking into account different information about the patient. The introduction of secular trend substantially changed individual risk predictions. The largest uncertainty in individual risk prediction occurred in patients with higher risks (i.e. those who are considered for statin treatment) with a large number of patients being reclassified as no longer requiring statin treatment.

The QRISK models did not consider the secular trend, and their follow-up was also restricted to more historic data (starting in 1998 [43]). In the present study, the largest contributing factor to the within-person variability in the CVD estimates was the secular trend. After introducing the secular trend into the modelling, 62% of females and 51% of males in 2016 would be classified down from a CVD risk ≥ 10% to less than 10% risk and thus no longer be eligible for statin treatment according to guidelines. When extrapolating to the population in England, this could affect almost 4 million individuals. Other studies have also reported a reduction in the CVD incidence over time [22, 44, 45]. A nation-wide study in England reported that the rate of hospitalisations for acute myocardial infarction reduced by 5% annual between 2002 and 2010, which is similar to our estimates [44]. Better CVD prevention may have contributed to this decline, which could include an increase in statin use [46]. Given the use of these models is mandated in NICE guidelines, it is quite likely this is caused by QRISK resulting in a prediction paradox [47], and the increase in statin use could explain this secular trend. However our analyses found that the cohort of statin users also showed a decreased CVD risk over time, suggesting that other factors may have contributed to the decline in CVD incidence. It is important that clinicians and patients are made aware of this as inclusion of the secular trend into the QRISK models could massively reduce the number of patients who were eligible to receive treatment with statin therapy. There are many ways to address a secular trend in predictive models. The first is to re-calibrate the model to the time period of interest [9, 48], which is effectively what QRISK developers do by updating the time period in which they derive the model each year. However this still allows for a large un-modelled secular trend occurring between the study start and end date. This can also be done on a continuous scale using continuous model/Bayesian updating and can be used with a forgetting factor to down weight historical data [48]. However this also constitutes developing a model in some data, and updating it in light of new data, and therefore suffers the same problems. Varying coefficient models are also available which allow the relationship between predictors and outcomes to vary over time [48]. Our approach is equivalent to a special case of these models, where only the intercept is allowed to vary over time. The use of varying coefficient models to model the secular trend should be considered in future work, although a more detailed assessment of whether the secular trend is associated with changes in database usage, and the role of statin use on the secular trend would have to be carried out.

Other factors also contributed to non-negligible levels of variability in risk prediction, for example the effect of using mean imputation to impute patient data. This is relevant because we found there are missing data amongst the statin users’ cohort at statin initiation, which is the group of patients who should be having their risk assessed. For these patients, using mean imputation adds an avoidable level of uncertainty to the risk score. It is therefore important to measure all risk factors and include the measurements rather than relying on mean imputed values. Beyond this, we highlighted the variability in risk scores caused by introducing a variety of risk factors into the models. All factors that were introduced into the models have been shown in the literature to be risk factors of CVD [4, 13, 21, 22]. However there are many other factors that we could not evaluate, such as diet [49, 50], level of physical inactivity [51], an accurate measure of alcohol consumption, transaminase levels [52], C-reactive protein levels [53] or biomarkers and genetic information [54, 55]. This means the level of uncertainty associated with a risk score is likely to be far higher than what we have been able to highlight in this paper. Despite this, there is no feasible way for these risk factors to be incorporated into a model used at point of care in routine practice, as they are not routinely recorded. We are not trying to recommend the collection and inclusion of such factors to improve the current models used in practice. Rather, we have highlighted that the introduction of new risk factors that could be measured has a sizeable effect on individual risk, and this effect would be bigger if one were able to collect such risk factors and incorporate them also.

This study found that widely used population-level performance metrics of risk predictions were not very sensitive with varying modelling approaches in contrast to the individual risk predictions. Harrell’s *C* statistic [10] is the most commonly used performance metric but the comparisons between models showed marginal change. This finding is consistent with literature that reported that in well-performing models, *C* statistics are not sensitive to the introduction of new covariates [30, 56]. The measures of explained variation and randomness were more sensitive to the modelling decisions, mostly increasing by 0.2 across all the models. The *D* statistic showed the largest absolute increase, although this is unsurprising given it is not bounded by 0 and 1. While none of these metrics were developed to assess variability on the individual level, the large variability in individual risk but lack of variability in population-level performance metrics is of importance to the patient being treated. It should also be noted that there was a general trend of improved performance as variables were added to the models, potentially leading to the conclusion that adding any variable that may be associated with CVD will improve risk prediction. We do not believe this to be the case and think the trend is likely explained by increasing amounts of overfitting as more variables are added to the model. Although split sample techniques were used to derive the performance metrics, the sample is very large and the test data is likely to be representative of the development cohort. You therefore would expect improved performance as more variables were added when carrying out internal validation. National treatment guidelines in the UK state that ‘all CVD risk assessment tools can provide only an approximate value for CVD risk’ and that ‘interpretation of CVD risk scores should always reflect informed clinical judgement’ [4]. Our results highlight the importance of this, considering clinical judgement and supplementing these model estimates with evidence on additional risk factors. Despite this recommendation, our experience is that output from QRISK is regularly used to guide treatment decisions, while confusion remains around its interpretation [57]. Furthermore, there has been a recent push by Public Health England [58, 59] for self-assessment by the public of risk using a tool JBS3 [6] which is based on the lifetime QRISK model [60]. Arguably, patients will need to be informed about the approximate estimates of these tools and the need for clinical judgement. This is very much an issue about communication of the limitations of such estimates, rather than an issue with the models themselves. It may be important not to communicate a single value which does not take into account important risk factors such as diet, exercise and lifestyle [61], the severity of presenting comorbidities or the uncertainty underlying the modelling decisions.

There are several limitations in this study. While the dataset used to derive the models is similar to that used to derive QRISK3 in terms of demographics, there may be many other hidden differences between the datasets, for example geographical coverage or coding practices between the databases. This means our models do not directly represent the ones used in practice in England. One limitation was that a crude disease classification was used to derive many of the predictor variables. A combination of medical and/or prescription codes was used which may be sensitive to the choice of the code lists. Another limitation of this study was that important information on other risk factors was missing (such as diet or exercise), which could explain a large amount of unexplained variation in risk. Frailty models were considered to quantify the level of unexplained variation in patient risk due to missing covariates [62]. However we were unable to fit these models in a consistent fashion to the data, while also finding strong arguments against this methodology [63]. We also did not consider the variability in coding between practices, or between databases. Models may perform erroneously when used in a database in which it was not developed, an issue which has caused issues in recent history [12]. For example how will a model perform in a database that uses a different coding system? This was not considered in this study as data from two databases with different coding systems was not available; however this is an important area for future research. Finally, this paper focused on uncertainty induced by considering different information about the patient. However there may also be uncertainty associated with the risk scores caused by various modelling decisions. For example in models developed in this way, the target population is not well defined. The association of covariates with the outcome may change with age, and although interaction terms are included, it is difficult to truly model these relationships. Given these models are used to generate risk scores for patients over a wide age range, this could also induce uncertainty on the patient level. There are many other methodological choices which induce uncertainty, which should be explored in their own right. This paper focuses primarily on the choice of what information about the patients to include in the models.

## Conclusion

In conclusion, we found sizeable levels of uncertainty in the prediction of individual CVD risks for patients. Variations in the selection of covariates, inclusion of the secular trend in CVD incidence, geographical variation and different approaches to handling missing data considerably changed predictions. This high level of instability was not detected with conventional population-level model performance metrics. Extrapolating to the population in England, 3.8 million patients could be misclassified as requiring statin treatment depending on the model used, which is mostly down to the inclusion of the secular trend in CVD incidence. Population-level risk prediction models that are based on routinely collected data should not be used in isolation due to the uncertainty in the predictions. Clinical judgement, as recommended in national treatment guidelines [4], supplemented with evidence of additional risk factors, should be an essential part of individual decision-making. Uncertainty analyses with varying of modelling choices and quantification of incomplete evidence should routinely be conducted to assess uncertainty beyond the confidence interval.

## Additional files

Additional file 1: Predictor variable information and code list. More detailed information about the derivation of predictor variables is provided, as well as the code lists used to extract data from CPRD. (DOCX 641 kb)

Additional file 2: Density and convergence plots for imputed variables. We provide information about the imputation process and how we assessed the performance of the imputation. Convergence plots are provided to highlight mixing of Markov chains, and density plots compare imputed to non-imputed data. (DOCX 623 kb)

Additional file 3: TRIPOD checklist, prediction model development and validation. Description of data: self-assessment of TRIPOD reporting guidelines for model development and validation is provided. This is only applicable to aspects of the paper which involve model development or validation. (DOCX 91 kb)

Additional file 4: Supplementary tables. Contains supplementary tables referenced in the main text, and other information that may be of interest to the reader. (DOCX 75 kb)

Additional file 5: Code for analysis. Contains code to run the analysis. README.docx file has instructions on how to set up data before running analysis. (7Z 59 kb)

## Data Availability

The datasets generated and/or analysed during the current study are not publicly available as this would be a breach of the contract with CPRD. However it can be obtained by a separate application to CPRD after getting approval from Independent Scientific Advisory Committee (ISAC). To apply for data, follow the instructions here: https://www.cprd.com/research-applications. Code for the analyses is contained in Additional file 5. Code for cohort derivation is not provided, but methods mimic the clear steps for cohort derivation outlined in the methods section of the QRISK3 [15] paper. Combined with the code lists and extra information provided in Additional file 1, the steps for cohort derivation should be reproducible.

## Abbreviations

BMI: Body mass index
CKD: Chronic kidney disease
CPRD: Clinical Practice Research Datalink
CVD: Cardiovascular disease
EHR: Electronic health records
HDL: High-density lipoprotein
HES: Hospital episode statistics
HR: Hazard ratio
IBS: Integrated brier score
ONS: Office for National Statistics
SBP: Systolic blood pressure
SHA: Strategic health authority
THIN: The Health Improvement Network

## References

[1] Damen JAAG, Hooft L, Schuit E, Debray TPA, Collins GS, Tzoulaki I, et al. Prediction models for cardiovascular disease risk in the general population: systematic review. BMJ 2016;353(1 Pt 2):i2416. Available from: http://www.ncbi.nlm.nih.gov/pubmed/27184143%5Cn; http://www.pubmedcentral.nih.gov/articlerender.fcgi?artid=PMC4868251 10.1136/bmj.i2416 PMC4868251 27184143

[2] Goldstein BA, Navar AM, Pencina MJ, Ioannidis JPA (2017). Opportunities and challenges in developing risk prediction models with electronic health records data: a systematic review. J Am Med Informatics Assoc.

[3] Hippisley-Cox J, Coupland C, Vinogradova Y, Robson J, Minhas R, Sheikh A (2008). Predicting cardiovascular risk in England and Wales: prospective derivation and validation of QRISK2. BMJ..

[4] NICE. Cardiovascular disease: risk assessment and reduction, including lipid modification [Internet]. 2016 [cited 2018 May 3]. Available from: https://www.nice.org.uk/guidance/cg181/chapter/1-recommendations

[5] Public Health England. Heart Age Campaign [Internet]. [cited 2018 Sep 14]. Available from: https://campaignresources.phe.gov.uk/resources/campaigns/78-heart-age/resources

[6] Deanfield J, Sattar N, Simpson I, Wood D, Bradbury K, Fox K, et al. Joint British Societies’ consensus recommendations for the prevention of cardiovascular disease (JBS3). Heart. 2014;100:ii1–ii67. 10.1136/heartjnl-2014-305693 24667225

[7] Campbell D. Four in five adults at risk of early death, heart-age test shows [Internet]. [cited 2018 Sep 14]. Available from: https://www.theguardian.com/society/2018/sep/04/four-in-five-adults-at-risk-of-early-death-heart-age-test-shows

[8] Nolan T. The NHS heart age test will overload GPs who are already under huge pressure. Bmj [Internet]. 2018;3930(September):k3930. Available from: http://www.bmj.com/lookup/doi/10.1136/bmj.k3930 10.1136/bmj.k3930 30232081

[9] Steyerberg EW. Clinical prediction models: a practical approach to development, validation, and updating. New York: Springer; 2009.

[10] Harrell FEH, Lee KL, Mark DB (1996). Multivariable prognostic models: issues in developing models, evaluating assumptions and adequacy, and measuring and reducing errors. Stat Med.

[11] BBC Health. Statins alert over IT glitch in heart risk tool [Internet]. 2016 [cited 2016 Dec 1]. Available from: http://www.bbc.co.uk/news/health-36274791.

[12] Digitalhealth.net. QRisk2 in TPP “fixed” but up to 270,000 patients affected [Internet]. 2016 [cited 2016 Dec 1]. Available from: https://www.digitalhealth.net/2016/06/qrisk2-in-tpp-fixed-but-up-to-270000-patients-affected/.

[13] van Staa Tjeerd-Pieter, Gulliford Martin, Ng Edmond S.-W., Goldacre Ben, Smeeth Liam (2014). Prediction of Cardiovascular Risk Using Framingham, ASSIGN and QRISK2: How Well Do They Predict Individual Rather than Population Risk?. PLoS ONE.

[14] Hofer E. The uncertainty analysis of model results. Springer International Publishing; 2018.

[15] Hippisley-Cox J, Coupland C, Brindle P (2017). Development and validation of QRISK3 risk prediction algorithms to estimate future risk of cardiovascular disease: prospective cohort study. BMJ.

[16] Collins GS, Altman DG (2012). Predicting the adverse risk of statin treatment: an independent and external validation of an updated version of QRISK2. BMJ.

[17] Herrett E, Gallagher AM, Bhaskaran K, Forbes H, Mathur R, van Staa T (2015). Data resource profile: Clinical Practice Research Datalink (CPRD). Int J Epidemiol.

[18] Digital N. Hospital Episode Statistics [Internet]. [cited 2018 May 3]. Available from: https://digital.nhs.uk/data-and-information/data-tools-and-services/data-services/hospital-episode-statistics

[19] Office for National Statistics [Internet]. [cited 2018 May 3]. Available from: https://www.ons.gov.uk/

[20] Collins GS, Altman DG (2010). An independent and external validation of QRISK2 cardiovascular disease risk score: a prospective open cohort study. BMJ..

[21] Allgulander C (2016). Anxiety as a risk factor in cardiovascular disease. Curr Opin Psychiatry.

[22] Bhatnagar P, Wickramasinghe K, Williams J, Townsend N. Trends in the epidemiology of cardiovascular disease in the UK. Heart 2016;(0):1–8. Available from: http://heart.bmj.com/content/early/2015/05/06/heartjnl-2015-307516.full 10.1136/heartjnl-2016-309573 PMC5256396 27550425

[23] CVD Statistics - BHF UK Factsheet [Internet]. [cited 2018 Jul 24]. Available from: https://www.bhf.org.uk/research/heart-statistics.

[24] Peckham Stephen, Falconer Jane, Gillam Steve, Hann Alison, Kendall Sally, Nanchahal Kiran, Ritchie Benjamin, Rogers Rebecca, Wallace Andrew (2015). The organisation and delivery of health improvement in general practice and primary care: a scoping study. Health Services and Delivery Research.

[25] Van Buuren S, Groothuis-Oudshoon K. MICE: Multivariate Imputation by Chained Equations in R. J Stat Softw. 2011;45(3):1–67.

[26] Benner A. Multivariable fractional polynomials [Internet]. [cited 2018 Jul 24]. Available from: https://cran.r-project.org/web/packages/mfp/vignettes/mfp_vignette.pdf

[27] Marshall A, Altman DG, Holder RL, Royston P. Combining estimates of interest in prognostic modelling studies after multiple imputation: current practice and guidelines. BMC Med Res Methodol. 2009;9:57. 10.1186/1471-2288-9-57 PMC2727536 19638200

[28] Choodari-Oskooei B, Royston P, Parmar MKB (2012). A simulation study of predictive ability measures in a survival model I: explained variation measures. Stat Med.

[29] Rahman MS, Ambler G, Choodari-Oskooei B, Omar RZ (2017). Review and evaluation of performance measures for survival prediction models in external validation settings. BMC Medical Res Methodol.

[30] Austin PC, Pencinca MJ, Steyerberg EW (2017). Predictive accuracy of novel risk factors and markers: a simulation study of the sensitivity of different performance measures for the Cox proportional hazards regression model. Stat Methods Med Res.

[31] Uno H, Cai T, Pencina MJ, D’Agostino RB, Wei LJ (2011). On the C-statistics for evaluating overall adequacy of risk prediction procedures with censored survival data. Stat Med.

[32] Gönen M, Heller G (2005). Concordance probability and discriminatory power in proportional hazards regression. Biometrika.

[33] Royston Patrick, Sauerbrei Willi (2004). A new measure of prognostic separation in survival data. Statistics in Medicine.

[34] Kent John T., O'Quigley John (1988). Measures of Dependence for Censored Survival Data. Biometrika.

[35] O’Quigley J, Xu R, Stare J (2005). Explained randomness in proportional hazards models. Stat Med.

[36] Graf E, Schmoor C, Sauerbrei W, Schumacher M (1999). Assessment and comparison of prognostic classification schemes for survival data. Stat Med Stat Med.

[37] Gerds TA, Schumacher M (2006). Consistent estimation of the expected brier score in general survival models with right-censored event times. Biom J.

[38] Kent JT, O’Quigley J (1988). Measures of dependence for censored survival data. Biometrika..

[39] Royston P (2006). Explained variation for survival models. Stata J.

[40] Choodari-Oskooei B, Royston P, Parmar MKB (2012). A simulation study of predictive ability measures in a survival model II: explained randomness and predictive accuracy. Stat Med.

[41] ClinRisk. QRISK2 online calculator, Information, What is the difference between an “estimated” QRISK®2 CVD score and an “actual” QRISK®2 CVD score? [Internet]. [cited 2018 Dec 7]. Available from: https://qrisk.org/2017/index.php.

[42] Statistics O for N. Estimates of the population for the UK, England and Wales, Scotland and Northern Ireland [Internet]. 2018. Available from: https://www.ons.gov.uk/peoplepopulationandcommunity/populationandmigration/populationestimates/datasets/populationestimatesforukenglandandwalesscotlandandnorthernireland.

[43] ClinRisk. QRisk 2016 annual update information 2016. [Internet]. [cited 2018 Jul 24]. Available from: https://qrisk.org/2017/QRISK2-2016-Annual-Update-Information.pdf.

[44] Smolina K, Wright FL, Rayner M, Goldacre MJ (2012). Determinants of the decline in mortality from acute myocardial infarction in England between 2002 and 2010: linked national database study. BMJ.

[45] Lee S., Shafe A. C. E., Cowie M. R. (2011). UK stroke incidence, mortality and cardiovascular risk management 1999-2008: time-trend analysis from the General Practice Research Database. BMJ Open.

[46] Pajouheshnia R, Damen JAAG, Groenwold RHH, Moons KGM, Peelen LM. Treatment use in prognostic model research: a systematic review of cardiovascular prognostic studies. Diagnostic Progn Res BioMed Central. 2017;1(1):15. 10.1186/s41512-017-0015-0 PMC6460846 31093544

[47] Peek N, Sperrin M, Mamas M, van Staa T, Buchan I. Hari Seldon, QRISK3, and the prediction paradox [internet]. BMJ Responses. 2017; Available from: https://www.bmj.com/content/357/bmj.j2099/rr-0.

[48] Jenkins DA, Sperrin M, Martin GP, Peek N (2018). Dynamic models to predict health outcomes: current status and methodological challenges. Diagnostic Progn Res.

[49] Schulze MB, Martínez-González MA, Fung TT, Lichtenstein AH, Forouhi NG. Food based dietary patterns and chronic disease prevention. BMJ 2018;k2396. Available from: http://www.bmj.com/lookup/doi/10.1136/bmj.k2396 10.1136/bmj.k2396 PMC5996879 29898951

[50] Hooper L, Summerbell CD, Higgins JPT, Thompson RL, Capps NE, Smith GD (2001). Dietary fat intake and prevention of cardiovascular disease: systematic review. Br Med J.

[51] Brown WJ, Pavey T, Bauman AE (2015). Comparing population attributable risks for heart disease across the adult lifespan in women. Br J Sports Med.

[52] Weng Stephen F, Kai Joe, Guha Indra Neil, Qureshi Nadeem (2015). The value of aspartate aminotransferase and alanine aminotransferase in cardiovascular disease risk assessment. Open Heart.

[53] Libby P, Ridker PM, Maseri A (2002). Inflammation and atherosclerosis. Circulation..

[54] Berezin AE (2016). Biomarkers for cardiovascular risk in patients with diabetes. Heart..

[55] Dhingra Ravi, Vasan Ramachandran S. (2017). Biomarkers in cardiovascular disease: Statistical assessment and section on key novel heart failure biomarkers. Trends in Cardiovascular Medicine.

[56] Harrel F. Regression modeling strategies: with applications to linear models, logistic regression, and survival analysis. New York: Springer; 2001.

[57] Liew SM, Blacklock C, Hislop J, Glasziou P, Mant D (2013). Cardiovascular risk scores: qualitative study of how primary care practitioners understand and use them. Br J Gen Pract.

[58] BBC NEWS. How your heart age is key to heart attack or stroke risk [Internet]. [cited 2018 Sep 11]. Available from: https://www.bbc.co.uk/news/health-45395576.

[59] Petter O. How to check your heart age: three ways to determine the likelihood of a stroke or heart attack [Internet]. [cited 2018 Sep 11]. Available from: https://www.independent.co.uk/life-style/health-and-families/heart-age-check-health-public-health-england-stroke-a8522706.html

[60] Hippisley-Cox J, Coupland C, Robson J, Brindle P (2010). Derivation, validation, and evaluation of a new QRISK model to estimate lifetime risk of cardiovascular disease: cohort study using QResearch database. Bmj..

[61] Stewart Jack, Manmathan Gavin, Wilkinson Peter (2017). Primary prevention of cardiovascular disease: A review of contemporary guidance and literature. JRSM Cardiovascular Disease.

[62] Austin Peter C. (2017). A Tutorial on Multilevel Survival Analysis: Methods, Models and Applications. International Statistical Review.

[63] O’Quigley J, Stare J (2002). Proportional hazards models with frailties and random effects. Stat Med.

