# Correction to: The uncertainty with using risk prediction models for individual decision making: an exemplar cohort study examining the prediction of cardiovascular disease in English primary care

**DOI:** 10.1186/s12916-019-1404-8

**Published:** 2019-08-10

**Authors:** Alexander Pate, Richard Emsley, Darren M. Ashcroft, Benjamin Brown, Tjeerd van Staa

**Affiliations:** 1 Centre of Health eResearch, School of Health Sciences, Faculty of Biology, Medicine and Health, The University of Manchester, Oxford Road, Manchester, M13 9PL UK; 2 Department of Biostatistics and Health Informatics, Institute of Psychiatry, Psychology and Neuroscience, King’s College London, De Crispigny Park, London, SE5 8AF UK; 3 NIHR Greater Manchester Patient Safety Translational Research Centre, School of Health Sciences, Faculty of Biology, Medicine and Health, The University of Manchester, Oxford Road, Manchester, M13 9PL UK; 4 NIHR School for Primary Care Research, Centre for Primary Care, Division of Population of Health, Health Services Research and Primary Care, Manchester Academic Health Science Centre, University of Manchester, Manchester, M13 9PL UK; 5 Public Health England North West, 3 Piccadilly Place, London Road, Manchester, M1 3BN UK; 6 Division ofPharmacoepidemiology and Clinical Pharmacology, Utrecht Institute of Pharmaceutical Sciences, Utrecht University, Utrecht, Netherlands

**Correction to: BMC Med 17:134**

**https://doi.org/10.1186/s12916-019-1368-8**

The original article [1] contained an error in the abstract. The mentioned cohort size now correctly states ‘*N* = 3,855,660’.
