# Supplementary material for: The uncertainty with using risk prediction models for individual decision making: an exemplar cohort study examining the prediction of cardiovascular disease in English primary care
Source: BMC Med. 2019 Jul 17;17:134. doi: 10.1186/s12916-019-1368-8 (PMC6636064; doi:10.1186/s12916-019-1368-8)
Supplement: Supplementary file 1 — Predictor variable information and code list. More detailed information about the derivation of predictor variables is provided, as well as the code lists used to extract data from CPRD. (DOCX 641 kb) [file 12916_2019_1368_MOESM1_ESM.docx]

**S1 Appendix – Predictor variable information and code lists**

**Breakdown of predictor variables and how they were derived**

All ‘history of’ variables were derived looking back in the patients’ medical record from the index date for the codes from the relevant code list (below). For ethnicity the entire patients’ medical record was searched as this variable cannot change.

When deriving test data we followed QRISK methods wherever possible. For test data we looked as far back as five years prior to the index date. Standard deviation of systolic blood pressure was only recorded if a patient had two or more values in the previous five years, taking the standard deviation of all recorded values. For cholesterol and HDL, we also looked forward in time up until a CVD event or censored, or five years time. Drugs at baseline were defined as at least two prescriptions, with at least one in the 28 days before the index date.

The number of prescriptions in the last year was derived from the number of distinct prescription items per day (i.e. duplicates on the same day were removed, but both counted if on different days). Number of days with medical records was the number of distinct days with a Read code in the year prior to index date.

Severe mental illness included codes for depression, as is noted in the rapid responses section on the BMJ website: <https://www.bmj.com/content/357/bmj.j2099/rapid-responses>

Chronic Kidney Disease was calculated using read codes and an algorithm using test data. The algorithm uses eGFR scores and comes from this paper[1]. However many patients have creatinine recorded as opposed to eGFR. The recommended equation to convert creatinine to eGFR is the CKD-EPI[2] equation. This is recommended in the KDIGO guidelines[3], and this recent comparison[4] comparing CKD-EPI to the MDRD equation, which used to be the most commonly used equation. Therefore I extracted creatinine and eGFR scores in order to calculate presence of CKD using the above referenced algorithm.

**Full algorithms to calculate BMI, SBP, SBP variability, Cholesterol/HDL ratio, smoking status and CKD are available on request.**

Calendar time was calculated as the number of days from 1^st^ Jan 1998 that the patients index date was on.

The region variable used was the strategic health authority variable, recorded in CPRD.

**Code lists**

Code lists for the outcome variable, cardiovascular event, were available amongst the supplementary material of the QRISK3 paper published online. For all covariates that were included in QRISK2, code lists were available from the study by Van Staa et al[5]., which compared QRISK2, ASSIGN and Framingham. I then also used the code lists available from QOF as an alternative set of code lists, given I was not sure what had been used in the QRISK3 paper.

For variables not in QRISK2, or part of QOF, code lists were not available. This was anxiety, alcohol abuse, atypical anti-psychotic medication, erectile dysfunction, HIV/AIDS, left ventricular hypertrophy (LVH), migraine and systemic lupus erythematosus. For these codes were either generated through the CPRD code browser, or were available on the following websites:

<http://www.phpc.cam.ac.uk/pcu/cprd_cam/codelists/>[6]

<https://clinicalcodes.rss.mhs.man.ac.uk/>[7]

| **Variable** | **Code list (name of file in place where its read in to be converted to v3)** |
| --- | --- |
| Atrial Fibrilation | atrialfibqof_medcode   |
| Cholesterol | cholesterol_medcode   |
| CKD | chronicrenal345qof_medcode   |
| GFR | gfr_medcode   |
| CRP | crp_medcode   |
| Ethnicity | ethnicity_rohini_medcode   |
| Famhist | Famhis_lstrict_v2   |
| HDL | hdl_medcode   |
| Hypertension | hypertensionqof_medcode   |
| LDL HDL ratio | ldl_hdl_ratio_medcode   |
| LDL | ldl_medcode   |
| LVH | lvh_medcode   |
| Rheumatoid arthritis | raqof_medcode   |
| Smoking Status | smoking_medcode   |
| SBP | systolic_medcode   |
| Diabetes | t12diaqof_medcode    t1diaqof_medcode    t2diaqof_medcode   |
| Alcohol abuse | Alcohol_medcode (CPRDCAM_ALC095_MEDCODES)   |
| Anxiety | Anxiety_medcode (CPRDCAM_ANX021_MEDCODES)   |
| Depression | depressionqof_medcode   |
| Severe Mental Illness | sev_men_illqof_medcode   |
| Erectile dysfunction | Erectile_medcode   |
| SLE | SLE_medcode   |
| Migraine | migraine_medcode   |
| HIV | HIV_medcode (HIV AIDS medcodes)   |
| CVD QRISK | cvd_qrisk   |
| History of CVD | cvdhis_medcode   |
| CVD via HES | ICD9_CVD    ICD10_CVD   |

**Prescription codes:**

| **Variable** | **Code list** |
| --- | --- |
| Hypertension | Hypertension_rx   |
| Corticosteroid use | Corticosteroidv3_rx   |
| Erecetile dysfunction | Erectile_rx   |
| A typical anti-psychotic medication | Anti_psy_2ndgen_rx   |
| Depression | Depression_rx   |
| Schizophrenia/bipolar | Sev_men_ill_rx   |

**References**

[1] K. Jameson, S. Jick, K. W. Hagberg, B. Ambegaonkar, A. Giles, and D. O. Donoghue, “Prevalence and management of chronic kidney disease in primary care patients in the UK,” no. September, pp. 1110–1121, 2014.

[2] A. S. Levey, L. A. Stevens, C. Frcp, C. H. Schmid, Y. L. Zhang, A. F. C. Iii, H. I. Feldman, J. W. Kusek, P. Eggers, F. Van Lente, and T. Greene, “A New Equation to Estimate Glomerular Filtration Rate Andrew,” *Ann. Intern. Med.*, vol. 150, no. 9, pp. 604–612, 2009.

[3] O. Journal and I. Society, “KDIGO 2012 Clinical Practice Guideline for the Evaluation and Management of Chronic Kidney Disease,” vol. 3, no. 1, 2013.

[4] K. Matsushita, M. Woodward, T. H. Jafar, D. H. Smith, M. Tonelli, D. G. Warnock, and A. S. Levey, “Comparison of Risk Prediction Using the CKD-EPI Equation and the MDRD Study Equation for Estimated Glomerular Filtration Rate,” vol. 307, no. 18, pp. 1941–1951, 2012.

[5] T. P. Van Staa, M. Gulliford, E. S. W. Ng, B. Goldacre, and L. Smeeth, “Prediction of cardiovascular risk using framingham, ASSIGN and QRISK2: How well do they predict individual rather than population risk?,” *PLoS One*, vol. 9, no. 10, 2014.

[6] C. U. Primary Care Unit, “CPRD @ Cambridge - Code Lists.” [Online]. Available: http://www.phpc.cam.ac.uk/pcu/cprd_cam/codelists/. [Accessed: 24-Jan-2018].

[7] U. of M. Institute of Population Health, “ClinicalCodes.org.” [Online]. Available: https://clinicalcodes.rss.mhs.man.ac.uk/. [Accessed: 24-Jan-2018].
