# Supplementary material for: The uncertainty with using risk prediction models for individual decision making: an exemplar cohort study examining the prediction of cardiovascular disease in English primary care
Source: BMC Med. 2019 Jul 17;17:134. doi: 10.1186/s12916-019-1368-8 (PMC6636064; doi:10.1186/s12916-019-1368-8)
Supplement: Supplementary file 2 — Density and convergence plots for imputed variables. We provide information about the imputation process and how we assessed the performance of the imputation. Convergence plots are provided to highlight mixing of Markov chains, and density plots compare imputed to non-imputed data. (DOCX 623 kb) [file 12916_2019_1368_MOESM2_ESM.docx]

S2 Appendix – Density and convergence plots for imputed variables

Alex Pate

12/04/2018

The following document details the imputation process for imputing the dataset used to derive models A – F.

Contents

[Methods 2](#_Toc521333819)

[Results - Female Cohort 3](#_Toc521333820)

[BMI 3](#_Toc521333821)

[SBP 4](#_Toc521333822)

[SBP standard deviation 5](#_Toc521333823)

[Cholesterol 6](#_Toc521333824)

[HDL 7](#_Toc521333825)

[Smoking 8](#_Toc521333826)

[Ethnicity 10](#_Toc521333827)

[Results - Male Cohort 11](#_Toc521333828)

[BMI 11](#_Toc521333829)

[SBP 12](#_Toc521333830)

[SBP standard deviation 13](#_Toc521333831)

[Cholesterol 14](#_Toc521333832)

[HDL 15](#_Toc521333833)

[Smoking 16](#_Toc521333834)

[Ethnicity 17](#_Toc521333835)

# Methods

***Imputation methods***

We used multiple imputation by chained equations to impute missing data for body mass index (BMI), systolic blood pressure (SBP) and SBP variability, cholesterol, HDL, smoking status and ethnicity. The program used to impute the data was the R package MICE[1]. We imputed 20 datasets and carried out 20 iterations for each dataset. Variables included in the imputation models were all predictor variables from the final model (including interaction terms and fractional polynomials), Nelson Aalen estimate of the cumulative hazard at the time of event/censored and the censoring indicator. All continuous variables were imputed using predictive mean matching, and polytomous regression for categorical variables[1].

***Imputation assessment methods***

The density plots show assess whether there are any systematic differences in covariates for those with missing data and those without, i.e. supports the missing at random assumption. The convergence plots show the imputation had reached a steady state when we drew the values.

# Results - Female Cohort

## BMI

BMI convergence plot


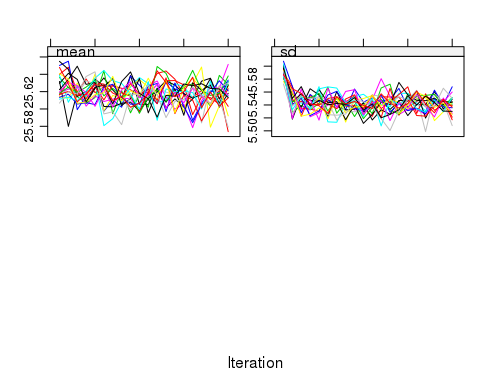


BMI density plot


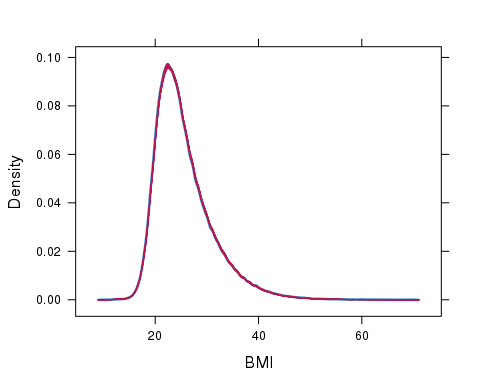


## SBP

SBP convergence plot


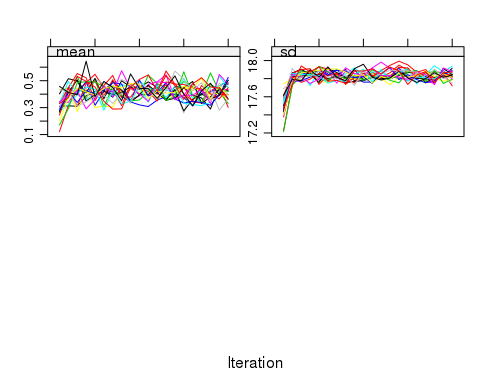


SBP density plot


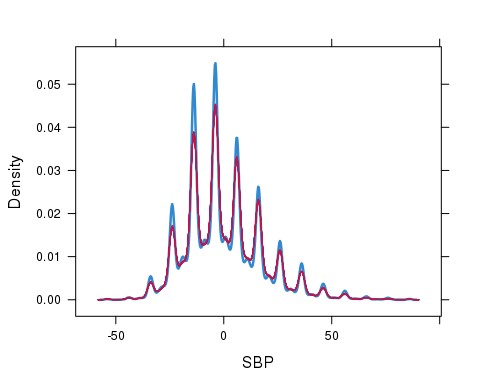


## SBP standard deviation

SBP standard deviation convergence plot


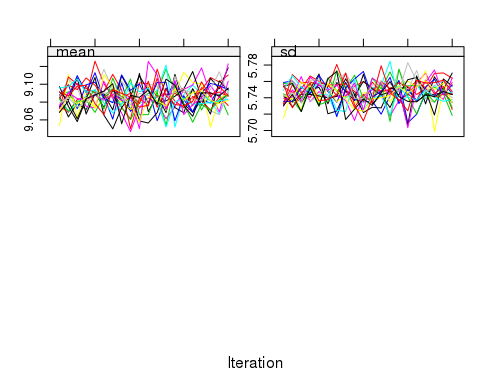


SBP standard deviation density plot


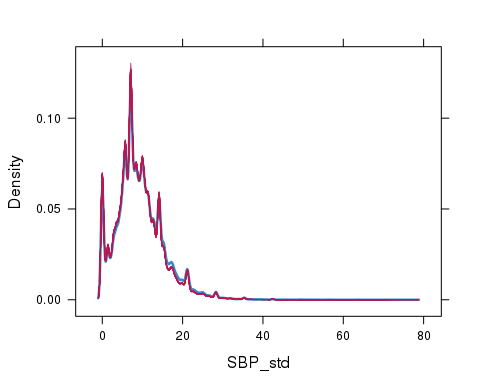


## Cholesterol

Cholesterol convergence plot


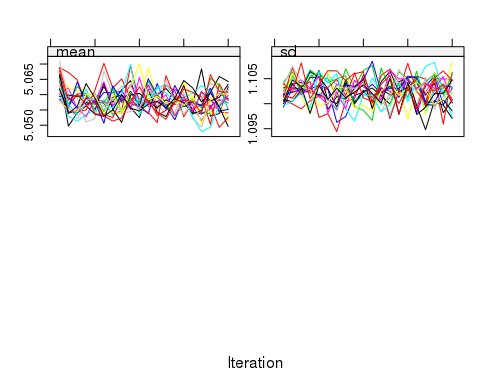


Cholesterol density plot


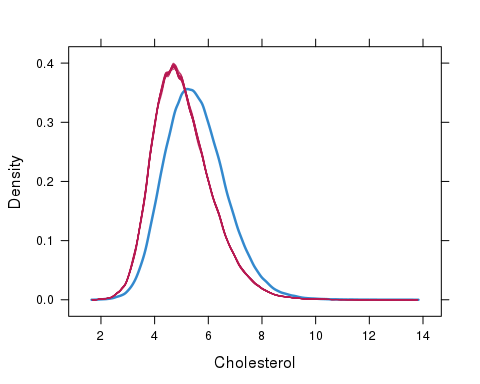


## HDL

HDL convergence plot


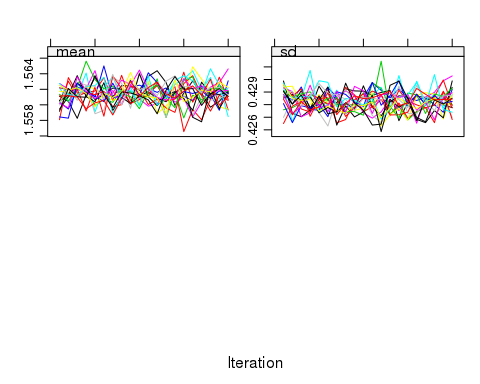


HDL density plot


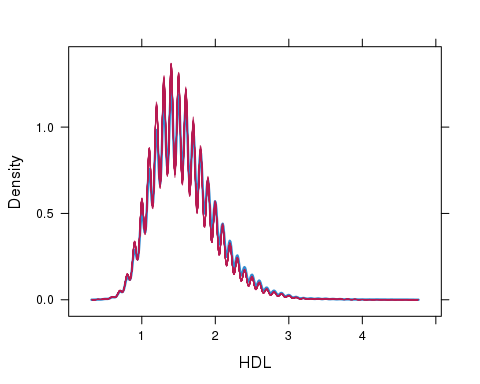


## Smoking

Distribution of non missing data

| Var1 | Freq |
| --- | --- |
| Never | 56.03 |
| Ex | 16.98 |
| Current | 27.00 |
|  |  |

Distribution of imputed data (by imputation)

|  | Never | Ex | Current |
| --- | --- | --- | --- |
| 1 | 57.92 | 16.15 | 25.93 |
| 2 | 58.33 | 16.27 | 25.40 |
| 3 | 58.78 | 15.76 | 25.47 |
| 4 | 57.50 | 15.96 | 26.55 |
| 5 | 59.48 | 15.92 | 24.61 |
| 6 | 58.98 | 16.12 | 24.90 |
| 7 | 57.65 | 15.90 | 26.45 |
| 8 | 57.94 | 15.77 | 26.29 |
| 9 | 58.33 | 15.71 | 25.96 |
| 10 | 59.18 | 15.70 | 25.12 |
| 11 | 58.90 | 16.05 | 25.05 |
| 12 | 59.06 | 15.45 | 25.49 |
| 13 | 58.35 | 15.77 | 25.88 |
| 14 | 57.90 | 16.19 | 25.91 |
| 15 | 58.62 | 15.78 | 25.60 |
| 16 | 58.56 | 16.00 | 25.44 |
| 17 | 58.84 | 15.69 | 25.46 |
| 18 | 59.23 | 15.11 | 25.66 |
| 19 | 58.50 | 15.65 | 25.85 |
| 20 | 59.45 | 15.56 | 25.00 |

## Ethnicity

| Imp | asianother | bangladesh | black | chinese | indian | mixed | oth_asian | other | pakistani | white |
| --- | --- | --- | --- | --- | --- | --- | --- | --- | --- | --- |
| Real data | 1.55 | 0.33 | 4.13 | 0.77 | 2.70 | 1.06 | 0.42 | 1.94 | 1.07 | 86.02 |
| 1 | 1.30 | 1.00 | 3.67 | 1.06 | 3.20 | 1.12 | 1.03 | 2.19 | 1.88 | 83.56 |
| 2 | 1.30 | 2.11 | 3.92 | 1.79 | 3.91 | 2.67 | 0.28 | 2.09 | 1.74 | 80.19 |
| 3 | 1.33 | 0.93 | 5.07 | 1.57 | 4.49 | 1.87 | 0.44 | 2.76 | 2.00 | 79.54 |
| 4 | 1.80 | 1.58 | 5.58 | 0.69 | 2.56 | 1.45 | 0.86 | 2.81 | 1.85 | 80.82 |
| 5 | 1.26 | 1.06 | 4.70 | 3.32 | 2.01 | 0.86 | 1.52 | 0.94 | 1.18 | 83.16 |
| 6 | 1.98 | 0.66 | 5.78 | 2.78 | 2.50 | 1.48 | 0.82 | 1.68 | 2.67 | 79.66 |
| 7 | 1.45 | 0.62 | 6.16 | 0.36 | 2.28 | 1.86 | 0.54 | 1.02 | 2.53 | 83.18 |
| 8 | 0.89 | 1.41 | 4.20 | 1.58 | 5.21 | 1.60 | 0.47 | 2.76 | 2.45 | 79.43 |
| 9 | 0.96 | 0.95 | 8.73 | 0.45 | 2.68 | 1.25 | 0.71 | 1.29 | 1.35 | 81.64 |
| 10 | 0.94 | 1.59 | 6.07 | 0.84 | 3.33 | 1.77 | 0.95 | 4.29 | 0.99 | 79.23 |
| 11 | 0.55 | 0.59 | 5.31 | 0.69 | 1.66 | 0.96 | 1.34 | 1.16 | 1.19 | 86.56 |
| 12 | 1.01 | 1.03 | 4.60 | 0.49 | 2.62 | 0.45 | 1.54 | 0.90 | 1.19 | 86.17 |
| 13 | 1.04 | 1.28 | 4.40 | 0.86 | 3.04 | 1.29 | 1.24 | 1.13 | 1.64 | 84.07 |
| 14 | 1.33 | 0.57 | 4.69 | 0.40 | 2.16 | 0.48 | 0.73 | 1.65 | 1.32 | 86.68 |
| 15 | 1.99 | 1.03 | 4.15 | 1.01 | 2.98 | 0.51 | 1.52 | 1.56 | 1.39 | 83.85 |
| 16 | 1.32 | 1.05 | 5.78 | 0.91 | 3.61 | 1.28 | 1.33 | 1.99 | 2.78 | 79.94 |
| 17 | 1.46 | 0.79 | 5.01 | 0.73 | 3.04 | 1.03 | 1.26 | 3.00 | 2.43 | 81.24 |
| 18 | 0.81 | 1.51 | 5.93 | 0.72 | 3.11 | 2.07 | 1.13 | 1.53 | 1.56 | 81.63 |
| 19 | 1.76 | 0.47 | 3.74 | 0.74 | 3.38 | 1.14 | 0.76 | 1.44 | 0.99 | 85.59 |
| 20 | 2.00 | 0.50 | 4.01 | 1.36 | 2.24 | 1.82 | 1.49 | 2.32 | 7.29 | 76.97 |

# Results - Male Cohort

## BMI

BMI convergence plot


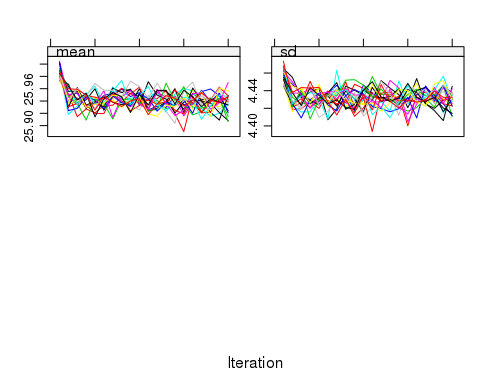


BMI density plot


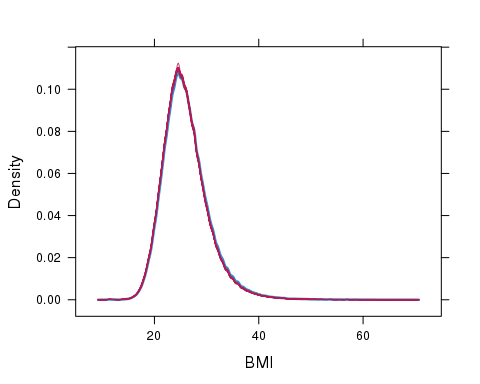


## SBP

SBP convergence plot


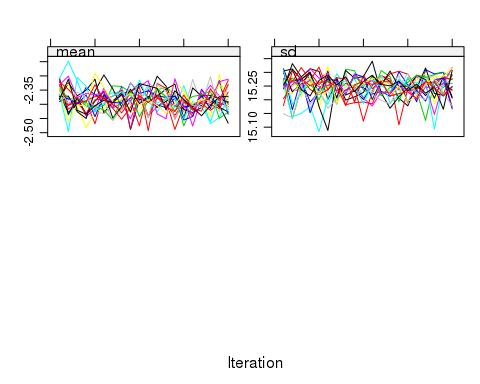


SBP density plot


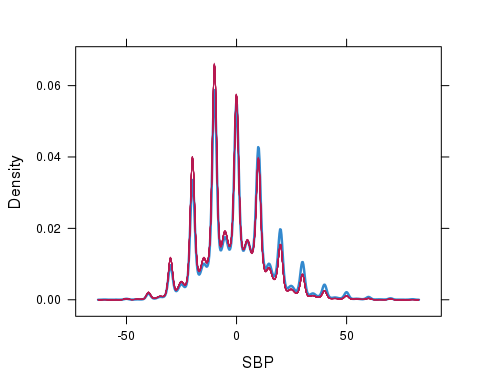


## SBP standard deviation

SBP standard deviation convergence plot


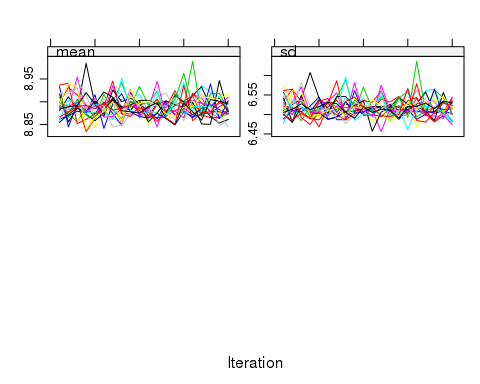


SBP standard deviation density plot


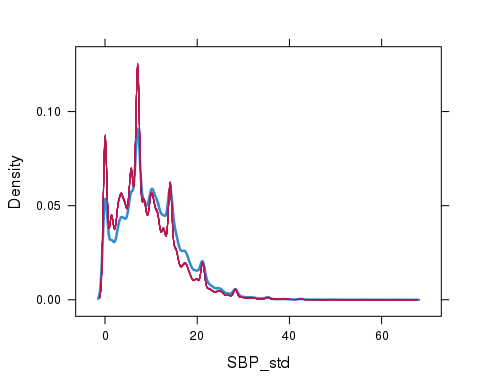


## Cholesterol

Cholesterol convergence plot


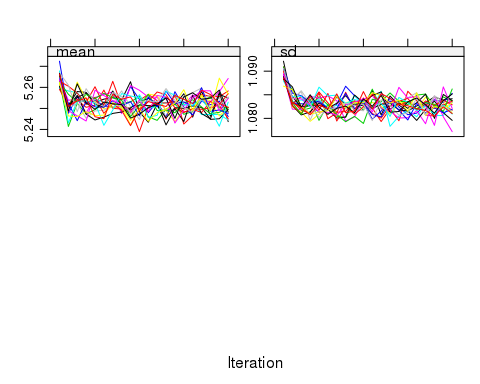


Cholesterol density plot


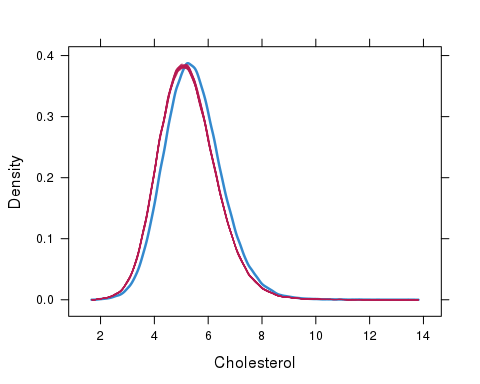


## HDL

HDL convergence plot


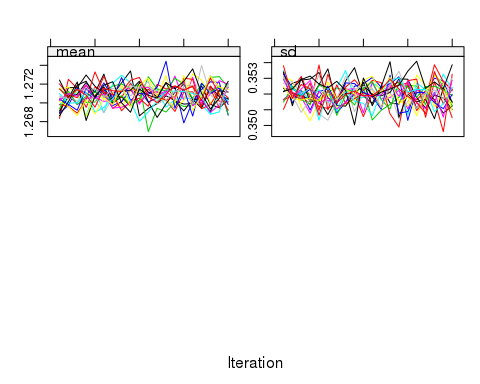


HDL density plot


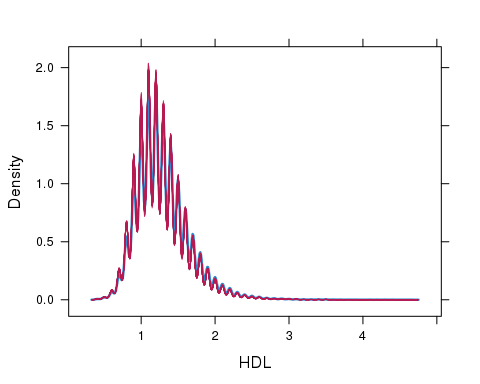


## Smoking

Distribution of non missing data

| Var1 | Freq |
| --- | --- |
| Never | 46.68 |
| Ex | 17.39 |
| Current | 35.93 |
|  |  |

Distribution of imputed data (by imputation)

| Imp | Never | Ex | Current |
| --- | --- | --- | --- |
| 1 | 48.44 | 17.29 | 34.27 |
| 2 | 48.65 | 17.20 | 34.15 |
| 3 | 48.83 | 17.05 | 34.12 |
| 4 | 48.20 | 17.51 | 34.29 |
| 5 | 48.35 | 17.24 | 34.40 |
| 6 | 48.31 | 17.34 | 34.35 |
| 7 | 48.23 | 17.27 | 34.50 |
| 8 | 48.28 | 17.34 | 34.39 |
| 9 | 48.20 | 17.36 | 34.44 |
| 10 | 48.76 | 17.03 | 34.22 |
| 11 | 48.43 | 17.18 | 34.39 |
| 12 | 48.18 | 17.30 | 34.51 |
| 13 | 47.90 | 17.35 | 34.75 |
| 14 | 48.32 | 17.45 | 34.23 |
| 15 | 48.37 | 17.36 | 34.27 |
| 16 | 48.62 | 17.21 | 34.18 |
| 17 | 48.43 | 17.21 | 34.35 |
| 18 | 48.21 | 17.42 | 34.37 |
| 19 | 48.16 | 17.24 | 34.60 |
| 20 | 49.21 | 17.06 | 33.70 |

## Ethnicity

| Imp | asianother | bangladesh | black | chinese | indian | mixed | oth_asian | other | pakistani | white |
| --- | --- | --- | --- | --- | --- | --- | --- | --- | --- | --- |
| Real data | 1.55 | 0.5 | 4.00 | 0.61 | 3.11 | 0.99 | 0.49 | 1.95 | 1.26 | 85.54 |
| 1 | 2.62 | 1.24 | 3.97 | 1.64 | 4.49 | 2.44 | 2.39 | 2.63 | 3.48 | 75.11 |
| 2 | 2.36 | 1.86 | 3.58 | 1.75 | 1.88 | 1.68 | 2.27 | 1.53 | 3.39 | 79.70 |
| 3 | 2.16 | 1.20 | 4.93 | 0.86 | 3.54 | 1.53 | 1.52 | 2.69 | 2.72 | 78.84 |
| 4 | 1.17 | 0.61 | 6.83 | 0.17 | 2.30 | 1.41 | 1.12 | 1.61 | 1.74 | 83.04 |
| 5 | 2.53 | 2.19 | 2.39 | 2.12 | 2.81 | 2.59 | 2.11 | 1.99 | 4.74 | 76.52 |
| 6 | 2.88 | 1.35 | 2.94 | 2.26 | 4.70 | 1.55 | 1.89 | 1.81 | 3.40 | 77.23 |
| 7 | 0.82 | 0.57 | 2.44 | 1.35 | 1.72 | 1.27 | 0.79 | 0.73 | 2.13 | 88.19 |
| 8 | 1.99 | 1.09 | 2.02 | 1.01 | 1.85 | 1.45 | 0.92 | 1.63 | 2.72 | 85.35 |
| 9 | 1.67 | 1.10 | 3.44 | 0.75 | 2.56 | 1.10 | 1.18 | 1.02 | 2.54 | 84.65 |
| 10 | 2.69 | 2.31 | 3.05 | 1.06 | 2.82 | 2.50 | 2.00 | 2.41 | 4.49 | 76.66 |
| 11 | 1.44 | 0.99 | 2.19 | 0.80 | 1.40 | 1.50 | 1.59 | 0.59 | 2.07 | 87.44 |
| 12 | 1.84 | 0.91 | 2.06 | 1.15 | 1.74 | 1.36 | 0.89 | 1.94 | 2.35 | 85.75 |
| 13 | 1.42 | 0.97 | 1.95 | 1.31 | 1.03 | 1.10 | 1.65 | 1.08 | 1.98 | 87.51 |
| 14 | 0.97 | 0.82 | 1.87 | 0.67 | 3.07 | 0.83 | 0.97 | 1.97 | 1.66 | 87.16 |
| 15 | 1.16 | 1.17 | 1.67 | 1.52 | 1.14 | 1.33 | 1.68 | 1.38 | 3.00 | 85.96 |
| 16 | 1.54 | 1.33 | 2.88 | 0.93 | 1.36 | 1.18 | 1.68 | 0.88 | 2.42 | 85.81 |
| 17 | 1.27 | 0.65 | 3.94 | 0.70 | 3.75 | 2.64 | 0.60 | 1.72 | 1.56 | 83.17 |
| 18 | 1.33 | 1.08 | 2.16 | 0.97 | 2.72 | 0.77 | 0.86 | 1.51 | 1.96 | 86.65 |
| 19 | 1.59 | 0.73 | 2.42 | 0.71 | 2.68 | 0.74 | 0.68 | 0.45 | 1.33 | 88.66 |
| 20 | 4.76 | 1.87 | 15.10 | 1.64 | 4.89 | 2.42 | 1.66 | 7.36 | 4.60 | 55.71 |
