# Supplementary material for: The uncertainty with using risk prediction models for individual decision making: an exemplar cohort study examining the prediction of cardiovascular disease in English primary care
Source: BMC Med. 2019 Jul 17;17:134. doi: 10.1186/s12916-019-1368-8 (PMC6636064; doi:10.1186/s12916-019-1368-8)
Supplement: Supplementary file 3 — TRIPOD checklist, prediction model development and validation. Description of data: self-assessment of TRIPOD reporting guidelines for model development and validation is provided. This is only applicable to aspects of the paper which involve model development or validation. (DOCX 91 kb) [file 12916_2019_1368_MOESM3_ESM.docx]

| **Section/Topic** | **Item** |  | **Checklist Item** | **Page** |
| --- | --- | --- | --- | --- |
| **Title and abstract** | | | | |
| Title | 1 | D;V | Identify the study as developing and/or validating a multivariable prediction model, the target population, and the outcome to be predicted.  The main objectives for this manuscript are not a model development + validation; therefore the title does not follow this format.  The manuscript does however involve the development and internal validation of a risk prediction model, and we report on the guidelines below for this process, where relevant. | 1 |
| Abstract | 2 | D;V | Provide a summary of objectives, study design, setting, participants, sample size, predictors, outcome, statistical analysis, results, and conclusions. | 3 |
| **Introduction** | | | | |
| Background and objectives | 3a | D;V | Explain the medical context (including whether diagnostic or prognostic) and rationale for developing or validating the multivariable prediction model, including references to existing models. | 5 |
|  | 3b | D;V | Specify the objectives, including whether the study describes the development or validation of the model or both.  The main objectives for this manuscript are not a model development + validation, therefore the objectives differ from what is requested here. | 6 |
| **Methods** | | | | |
| Source of data | 4a | D;V | Describe the study design or source of data (e.g., randomized trial, cohort, or registry data), separately for the development and validation data sets, if applicable. | 7 |
|  | 4b | D;V | Specify the key study dates, including start of accrual; end of accrual; and, if applicable, end of follow-up. | 7 |
| Participants | 5a | D;V | Specify key elements of the study setting (e.g., primary care, secondary care, general population) including number and location of centres. | 7 |
|  | 5b | D;V | Describe eligibility criteria for participants. | 7 |
|  | 5c | D;V | Give details of treatments received, if relevant. | NA |
| Outcome | 6a | D;V | Clearly define the outcome that is predicted by the prediction model, including how and when assessed. | 7 |
|  | 6b | D;V | Report any actions to blind assessment of the outcome to be predicted.  None – routinely collected data | NA |
| Predictors | 7a | D;V | Clearly define all predictors used in developing or validating the multivariable prediction model, including how and when they were measured. | 8/Appendix 3 . |
|  | 7b | D;V | Report any actions to blind assessment of predictors for the outcome and other predictors.  None – routinely collected data | NA |
| Sample size | 8 | D;V | Explain how the study size was arrived at.  This was all patients that met the eligibility criteria | 7 |
| Missing data | 9 | D;V | Describe how missing data were handled (e.g., complete-case analysis, single imputation, multiple imputation) with details of any imputation method. | 8/9/Appendix 2 |
| Statistical analysis methods | 10a | D | Describe how predictors were handled in the analyses. | 8/9 |
|  | 10b | D | Specify type of model, all model-building procedures (including any predictor selection), and method for internal validation.  Classical predictor selection was not carried out (i.e. backwards selection) as we pre-specified different sets of predictors for each model, this was a key part of the study. | 8/9 |
|  | 10c | V | For validation, describe how the predictions were calculated. | 10 |
|  | 10d | D;V | Specify all measures used to assess model performance and, if relevant, to compare multiple models. | 10 |
|  | 10e | V | Describe any model updating (e.g., recalibration) arising from the validation, if done. | NA |
| Risk groups | 11 | D;V | Provide details on how risk groups were created, if done. | 12 |
| Development vs. validation | 12 | V | For validation, identify any differences from the development data in setting, eligibility criteria, outcome, and predictors.  *Rather than comparing the development and validation cohorts (which were chosen at random from the same cohort), we chose to compare the development cohort with the one from the published QRISK algorithm. This is more important for this study as it helps validate the generalisability of the results to the models used in practice. This is not meant to be a classical model development project.* The table was however very large given we had separate male and female cohorts, and therefore only the CPRD cohort is presented in the main text, with the comparison presented in Appendix 1 – Supplementary Table 1. | Table 1/Supplementary Table 1 |
| **Results** | | | | |
| Participants | 13a | D;V | Describe the flow of participants through the study, including the number of participants with and without the outcome and, if applicable, a summary of the follow-up time. A diagram may be helpful. | 14 |
|  | 13b | D;V | Describe the characteristics of the participants (basic demographics, clinical features, available predictors), including the number of participants with missing data for predictors and outcome. | , Table 1 |
|  | 13c | V | For validation, show a comparison with the development data of the distribution of important variables (demographics, predictors and outcome).  Validation was not the main aim of this paper, all comparisons have been done with QRISK, which is the model used in practice across the UK. See point 12. | Supplementary Table 1 |
| Model development | 14a | D | Specify the number of participants and outcome events in each analysis. | Table 1 |
|  | 14b | D | If done, report the unadjusted association between each candidate predictor and outcome. | NA |
| Model specification | 15a | D | Present the full prediction model to allow predictions for individuals (i.e., all regression coefficients, and model intercept or baseline survival at a given time point). | Table 2/Supplementary Table 6 |
|  | 15b | D | Explain how to the use the prediction model. | NA |
| Model performance | 16 | D;V | Report performance measures (with CIs) for the prediction model.  Given the size of the cohort performance metrics took a long time to derive. Confidence intervals for majority of these metrics can only be obtained by bootstrapping. This would involve deriving the metrics hundreds of times, which could take a lot of computational time. Given the size of the cohort I expect the confidence interval to be small and therefore I have not done this. | Table 3 |
| Model-updating | 17 | V | If done, report the results from any model updating (i.e., model specification, model performance). | NA |
| **Discussion**  **The discussion has a very different structure as the main aim of this paper was not development and validation of a model to be used in practice** | | | | |
| Limitations | 18 | D;V | Discuss any limitations of the study (such as nonrepresentative sample, few events per predictor, missing data). | 27 |
| Interpretation | 19a | V | For validation, discuss the results with reference to performance in the development data, and any other validation data. | NA |
|  | 19b | D;V | Give an overall interpretation of the results, considering objectives, limitations, results from similar studies, and other relevant evidence. | 24/25/26 |
| Implications | 20 | D;V | Discuss the potential clinical use of the model and implications for future research. | NA |
| **Other information** | | | | |
| Supplementary information | 21 | D;V | Provide information about the availability of supplementary resources, such as study protocol, Web calculator, and data sets. | 31 |
| Funding | 22 | D;V | Give the source of funding and the role of the funders for the present study. | 31 |

*Items relevant only to the development of a prediction model are denoted by D, items relating solely to a validation of a prediction model are denoted by V, and items relating to both are denoted D;V. We recommend using the TRIPOD Checklist in conjunction with the TRIPOD Explanation and Elaboration document.
