# Supplementary material for: The uncertainty with using risk prediction models for individual decision making: an exemplar cohort study examining the prediction of cardiovascular disease in English primary care
Source: BMC Med. 2019 Jul 17;17:134. doi: 10.1186/s12916-019-1368-8 (PMC6636064; doi:10.1186/s12916-019-1368-8)
Supplement: Supplementary file 4 — Supplementary tables. Contains supplementary tables referenced in the main text, and other information that may be of interest to the reader. (DOCX 75 kb) [file 12916_2019_1368_MOESM4_ESM.docx]

S4 Appendix – Supplementary Tables

**Main paper: The uncertainty with using risk prediction models for individual decision making: an exemplar cohort study examining the prediction of cardiovascular disease in English primary care**

Contents

[Table s1 - Baseline characteristics of study population and comparison with cohort used to derive QRISK3 2](#_Toc9516965)

[Table s2.1 – Incidence rates of CVD from CPRD cohort and comparison with QRISK3 by age band (female cohort) 5](#_Toc9516966)

[Table s2.2 – Incidence rates of CVD from CPRD cohort and comparison with QRISK3 by age band (male cohort) 6](#_Toc9516967)

[Table s3.1 - Comparison of hazard ratios of categorical variables from model B with QRISK3 7](#_Toc9516968)

[Table s3.2 – Hazard ratios of continuous variables from model B, associated with one unit increase 9](#_Toc9516969)

[Table s4: Numbers and percentages of patients registered on 1^st^ Jan 2016 who cross the treatment threshold (10 year CVD risk of 10%) when using models B –F instead of model A. 10](#_Toc9516970)

[Table s4 additional text - Breakdown of calculations for extrapolation to UK population 12](#_Toc9516971)

[References 14](#_Toc9516972)

# Table s1 - Baseline characteristics of study population and comparison with cohort used to derive QRISK3

|  | **QRESEARCH FEMALE**  **N=4019956** | **CPRD FEMALE**  **N=1965078** | **QRESEARCH MALE**  **N=3869847** | **CPRD MALE**  **N=1890582** |
| --- | --- | --- | --- | --- |
| **Outcome variables** | | | | |
| Incident CVD cases | 160549 | 86547 | 203016 | 107051 |
| Person years | 25943236 | 13801919 | 24821632 | 12977235 |
| Rate per 1000 person years | 6.19 | 6.27 | 8.18 | 8.24 |
| **Demographics \| mean (sd), for continuous** | | | | |
| Age | 43.3 (15.3) | 43.07 (15.94) | 42.6 (14.0) | 41.84 (14.57) |
| **Ethnicity:** Recorded | 64.9% | 42.07% | 59.7% | 38.21% |
| White/not recorded | 88.8% | 94.12% | 88.7% | 94.48% |
| Indian | 1.9% | 1.14% | 2.1% | 1.19% |
| Pakistani | 1.0% | 0.45% | 1.2% | 0.49% |
| Bangladeshi | 0.8% | 0.14% | 1.1% | 0.19% |
| Other Asian | 1.3% | 0.84% | 1.2% | 0.78% |
| Black Caribbean | 0.9% | Black = 1.73% | 0.8% | 1.52%  0.23% |
| Black African | 1.9% |  | 1.8% |  |
| Chinese | 0.8% | 0.33% | 0.6% | 1.12% |
| Other | 2.6% | 1.27 % (includes mixed race) | 2.4% | 38.21% |
| **Test data \| mean (sd), %recorded, for continuous** | | | | |
| BMI | 25.4 (5.1), 72.8% | 25.60 (5.60), 68.83% | 25.9 (4.2), 64% | 26.12 (4.54), 53.62% |
| Cholesterol/HDL ratio | 3.7 (1.2), 39.8% | 3.72 (1.20), 38.48% | 4.4 (1.4), 37.9% | 4.48 (1.40), 35.71% |
| SBP | 123.2 (18.2), 82.8% | 123.91 (18.28), 81.01% | 129.2 (16.3), 68.3% | 130.03 (16.48), 59.21% |
| SBP variability | 9.3 (6.2), 77.7% | 9.47 (5.98), 50.39% | 9.9 (6.8), 64.0% | 10.13 (6.80), 20.94% |
| Smoking status | Never = 60%  Ex = 17.3%  Current = 22.7%  Recorded = 85% | Never = 56.04%  Ex = 16.97%  Current = 26.99%  Recorded = 75.18% | Never = 48.6%  Ex = 19.8%  Current = 31.5%  Recorded = 77.7% | Never = 46.63%  Ex = 17.48%  Current = 35.99%  Recorded = 65.17% |
| **Medical History** | | | | |
| Atrial Fibrillation | 0.4% | 0.44% | 0.5% | 0.57% |
| Atypical antipsychotic medication use | 0.5% | 0.30% | 0.5% | 0.33% |
| Chronic Kidney Disease  stage 3/4/5 | 0.5% | 0.45% | 0.3% | 0.32% |
| stage 4/5 | 0.2% | 0.12% | 0.2% | 0.15% |
| Corticosteroid use | 2.4% | 0.48% | 1.5% | 0.30% |
| Erectile dysfunction | NA | NA | 2.3% | 1.45% |
| Family history of CVD | 12% | 15.08% | 9.3% | 11.02% |
| HIV/AIDS | 0.1% | 0.06% | 0.2% | 0.09% |
| Migraine | 6.4% | 7.27% | 2.7% | 2.94% |
| Rheumatoid arthritis | 1.1% | 0.69% | 0.5% | 0.26% |
| Severe Mental Illness | 6.8% | 8.63% | 4.3% | 4.59% |
| Systemic Lupus Erythematosus | 0.1% | 0.10% | 0.0% | 0.01% |
| Treated hypertension | 5.6% | 6.18% | 4.2% | 4.50% |
| Type 1 diabetes | 0.3% | 0.21% | 0.3% | 0.28% |
| Type 2 diabetes | 1.2% | 1.16% | 1.5% | 1.42% |
| **Variables not in QRISK** | | | | |
| Number medical records in previous year | NA | 14.94 (13.97) | NA | 8.83 (11.45) |
| > 50 medical records in previous year | NA | 2.84% | NA | 1.37% |
| Number of prescription items in previous year | NA | 9.60 (19.87) | NA | 5.72 (16.00) |
| Number with > 50 prescription items in previous year | NA | 3.49% | NA | 2.04% |
| Alcohol abuse | NA | 0.65% | NA | 1.46% |
| Anxiety | NA | 13.44% | NA | 7.96% |
| Left Ventricular Hypertrophy | NA | 0.14% | NA | 0.18% |
| **Region*:** North East | NA | 1.89% | NA | 1.96% |
| North west | NA | 13.10% | NA | 13.38% |
| Yorkshire and the Humber | NA | 3.93% | NA | 3.85% |
| East Midlands | NA | 3.14% | NA | 3.23% |
| West Midlands | NA | 11.04% | NA | 11.28% |
| East of England | NA | 11.67% | NA | 11.68% |
| South west | NA | 11.99% | NA | 11.88% |
| South Central | NA | 12.84% | NA | 12.81% |
| London | NA | 17.52% | NA | 17.18% |
| South East Coast | NA | 12.88% | NA | 12.74% |

# Table s2.1 – Incidence rates of CVD from CPRD cohort and comparison with QRISK3 by age band (female cohort)

| CPRD cohort | | | |  | QRISK3 cohort | | | |
| --- | --- | --- | --- | --- | --- | --- | --- | --- |
| Age | Incident cases | Person years | Rate per 1000 person years |  | Age | Incident cases | Person years | Rate per 1000 person years |
| 25-29 | 626 | 2499863 | 0.25 |  | 25-29 | 832 | 3455662 | 0.24 |
| 30-34 | 1026 | 1777476 | 0.58 |  | 30-34 | 1878 | 3802577 | 0.49 |
| 35-39 | 1973 | 1739617 | 1.13 |  | 35-39 | 3636 | 3551460 | 1.02 |
| 40-44 | 3098 | 1507321 | 2.06 |  | 40-44 | 5651 | 2971995 | 1.9 |
| 45-49 | 4304 | 1342797 | 3.21 |  | 45-49 | 8272 | 2581104 | 3.2 |
| 50-54 | 6481 | 1281258 | 5.06 |  | 50-54 | 12022 | 2490263 | 4.83 |
| 55-59 | 7845 | 1013648 | 7.74 |  | 55-59 | 14524 | 1944140 | 7.47 |
| 60-64 | 9941 | 823913 | 12.07 |  | 60-64 | 18471 | 1625795 | 11.4 |
| 65-69 | 12374 | 678773 | 18.23 |  | 65-69 | 22510 | 1314303 | 17.1 |
| 70-74 | 13833 | 525681 | 26.31 |  | 70-74 | 25462 | 1015263 | 25.1 |
| 75-79 | 14134 | 390288 | 36.21 |  | 75-79 | 26883 | 765681 | 35.1 |
| 80-84 | 10912 | 221282 | 49.31 |  | 80-84 | 20408 | 424994 | 48.0 |
| Total | 86547 | 13801919 | 6.27 |  | Total | 160549 | 25943236 | 6.19 |
| Average observed risk | | 6.0% | |  | Average observed risk | | 5.8% | |
| Average predicted risk | | 5.0% | |  | Average predicted risk | | 4.7% | |

# Table s2.2 – Incidence rates of CVD from CPRD cohort and comparison with QRISK3 by age band (male cohort)

| CPRD cohort | | | |  | QRISK3 cohort | | | |
| --- | --- | --- | --- | --- | --- | --- | --- | --- |
| Age | Incident cases | Person years | Rate per 1000 person years |  | Age | Incident cases | Person years | Rate per 1000 person years |
| 25-29 | 906 | 2548923 | 0.36 |  | 25-29 | 1351 | 3379716 | 0.4 |
| 30-34 | 2012 | 1748443 | 1.15 |  | 30-34 | 3823 | 3880890 | 0.99 |
| 35-39 | 4245 | 1788096 | 2.37 |  | 35-39 | 7963 | 3748285 | 2.12 |
| 40-44 | 6811 | 1560256 | 4.37 |  | 40-44 | 12750 | 3192048 | 3.99 |
| 45-49 | 9826 | 1346772 | 7.30 |  | 45-49 | 17763 | 2972642 | 6.65 |
| 50-54 | 13036 | 1225972 | 10.63 |  | 50-54 | 24040 | 2437106 | 9.86 |
| 55-59 | 13637 | 911484 | 14.96 |  | 55-59 | 25464 | 1796342 | 14.18 |
| 60-64 | 14097 | 685171 | 20.57 |  | 60-64 | 27021 | 1372104 | 19.69 |
| 65-69 | 14111 | 507205 | 27.82 |  | 65-69 | 26903 | 1013291 | 26.55 |
| 70-74 | 12573 | 344401 | 36.51 |  | 70-74 | 24549 | 691866 | 35.48 |
| 75-79 | 9976 | 213435 | 46.74 |  | 75-79 | 19820 | 438864 | 45.16 |
| 80-84 | 5821 | 97078 | 59.96 |  | 80-84 | 11569 | 198481 | 58.29 |
| Total | 107051 | 12977235 | 8.24 |  | Total | 203016 | 24821632 | 8.18 |
| Average observed risk | | 7.7% | |  | Average observed risk | | 7.5% | |
| Average predicted risk | | 6.5% | |  | Average predicted risk | | 6.4% | |

# Table s3.1 - Comparison of hazard ratios of categorical variables from model B with QRISK3

|  | Female | | Male | |
| --- | --- | --- | --- | --- |
|  | CPRD cohort (model B) | QRISK3 | CPRD cohort (model B) | QRISK3 |
| Atrial fibrilation | 3.29 | 4.92 | 2.16 | 2.42 |
| Atypical antipsychotic medication use | 1.33 | 1.29 | 1.17 | 1.14 |
| Corticosteroid Use | 2.22 | 1.81 | 1.93 | 1.58 |
| CKD (stage 3/4/5) | 1.96 | 1.93 | 2.02 | 2.05 |
| Erectile dysfunction | NA | NA | 1.08 | 1.25 |
| Ethnicity:asianother | 1.37 | 1.08 | 1.13 | 1.04 |
| Ethnicity:bangladeshi | 1.30 | 1.34 | 1.16 | 1.70 |
| Ethnicity:black (African/Caribbean) | 1.35 | 0.84/0.67 | 0.95 | 0.70/0.67 |
| Ethnicity:chinese | 1.32 | 0.722 | 1.01 | 0.66 |
| Ethnicity:indian | 1.29 | 1.32 | 1.18 | 1.32 |
| Ethnicity:mixed | 1.51 | NA | 1.09 | NA |
| Ethnicity:other | 1.38 | 0.84 | 1.09 | 0.76 |
| Ethnicity:pakistani | 1.46 | 1.76 | 1.19 | 1.61 |
| Family history of CVD | 1.41 | 1.58 | 1.45 | 1.72 |
| Hypertension (treated) | 1.35 | 1.66 | 1.32 | 1.68 |
| Migraine | 1.23 | 1.35 | 1.19 | 1.29 |
| Rheumatoid arthritis | 1.42 | 1.24 | 1.35 | 1.23 |
| Severe mental illness | 1.30 | 1.13 | 1.22 | 1.13 |
| Smoker (Ex) | 1.28 | 1.14 | 1.19 | 1.21 |
| Smoker (current \| light/moderate/heavy) | 1.96 | 1.75/1.95/2.34 | 1.84 | 1.74/1.89/2.20 |
| Systemic lupus erythematosus | 1.55 | 2.14 | 1.10 | 1.55 |
| Townsend = 2 | 1.10 | NA | 1.03 | NA |
| Townsend = 3 | 1.27 | NA | 1.13 | NA |
| Townsend = 4 | 1.47 | NA | 1.26 | NA |
| Townsend = 5 (most deprived) | 1.83 | NA | 1.40 | NA |
| Type 1 diabetes | 4.29 | 5.62 | 2.88 | 3.44 |
| Type 2 diabetes | 2.92 | 2.91 | 2.30 | 2.36 |

Given we derived different fractional polynomials than those in QRISK there is no point comparing hazard ratios directly. The other continuous variables were also not modelled on the same scale meaning there is no point comparing hazard ratios of these. We report and transformations and fractional polynomials calculated from variables and the associated hazard ratios from model B.

**Transformations male cohort:**

Age.t = (age – 24.95)/10

BMI.t = BMI/10

SBP.t = SBP/100

**Fractional polynomials for the male cohort:**

Agefrac1 = age.t^2

Agefrac2 = age.t^0.5

**Transformations female cohort:**

Age.t = (age – 24.95)/10

BMI.t = BMI/10

SBP.t = SBP/100

**Fractional polynomials for the female cohort:**

Agefrac1 = age.t^0.5

Agefrac2 = log(age.t)*(age.t)^0.5

BMIfrac1 = (BMI.t)^-2

BMIfrac2 = log(BMI.t)*(BMI.t)^-2

# Table s3.2 – Hazard ratios of continuous variables from model B, associated with one unit increase

|  | Female | Male |
| --- | --- | --- |
|  | CPRD cohort (model B) | CPRD cohort (model B) |
| Agefrac1 | 2.83 | 1.01 |
| Agefrac2 | 2.12 | 14.66 |
| BMI | NA | 1.14 |
| BMIfrac1 | 1.50 | NA |
| BMIfrac2 | 0.01 | NA |
| SBP | 3.65 | 4.24 |
| SBP standard deviation | 1.01 | 1.01 |
| Cholesterol/HDL ratio | 1.14 | 1.15 |

# Table s4: Numbers and percentages of patients registered on 1^st^ Jan 2016 who cross the treatment threshold (10 year CVD risk of 10%) when using models B –F instead of model A.

|  | Predicted CVD risk according to model A (QRISK2) | | | | | | Predicted CVD risk according to model A (QRISK2) | | | | | |
| --- | --- | --- | --- | --- | --- | --- | --- | --- | --- | --- | --- | --- |
|  | 5-6% | 6-7% | 7-8% | 8-9% | 9-10% | **(<10%)** | 10-11% | 11-12% | 12-13% | 13-14% | 14-15% | **(>10%)** |
| **Female (N = 387547)** | | | | | | | | | | | | |
| N | 16148 | 13183 | 10816 | 9405 | 8180 | **317387** | 7387 | 6682 | 5935 | 5170 | 4684 | **70160** |
| Model B | 144 (1%) | 391 (3%) | 724 (7%) | 1582 (17%) | 2435 (30%) | **5362 (2%)** | 3129 (42%) | 373 (6%) | 29 (0%) | 15 (0%) | 9 (0%) | **3575 (5%)** |
| Model C | 118 (1%) | 263 (2%) | 582 (5%) | 1198 (13%) | 2159 (26%) | **4363 (1%)** | 3863 (52%) | 975 (15%) | 134 (2%) | 26 (1%) | 14 (0%) | **5064 (7%)** |
| model D | 4 (0%) | 6 (0%) | 18 (0%) | 27 (0%) | 43 (1%) | **98 (0%)** | 7322 (99%) | 6583 (99%) | 5804 (98%) | 4970 (96%) | 4440 (95%) | **43822 (62%)** |
| model E | 5 (0%) | 10 (0%) | 19 (0%) | 36 (0%) | 42 (1%) | **112 (0%)** | 7329 (99%) | 6581 (98%) | 5789 (98%) | 4965 (96%) | 4432 (95%) | **44826 (64%)** |
| model F | 7 (0%) | 13 (0%) | 22 (0%) | 45 (0%) | 58 (1%) | **147 (0%)** | 7313 (99%) | 6561 (98%) | 5762 (97%) | 4928 (95%) | 4404 (94%) | **44761 (64%)** |
| **Male (N = 352014)** | | | | | | | | | | | | |
| N | 18974 | 16897 | 14601 | 13211 | 11208 | **254714** | 9690 | 8668 | 7462 | 6737 | 6004 | **97300** |
| Model B | 64 (0%) | 152 (1%) | 333 (2%) | 988 (7%) | 2300 (21%) | **3859 (2%)** | 2696 (28%) | 154 (2%) | 5 (0%) | 1 (0%) | 0 (0%) | **2856 (3%)** |
| Model C | 55 (0%) | 202 (1%) | 432 (3%) | 1055 (8%) | 2489 (22%) | **4250 (2%)** | 3305 (34%) | 319 (4%) | 17 (0%) | 6 (0%) | 3 (0%) | **3652 (4%)** |
| model D | 2 (0%) | 5 (0%) | 8 (0%) | 20 (0%) | 55 (0%) | **91 (0%)** | 9610 (99%) | 8507 (98%) | 7224 (97%) | 6349 (94%) | 5481 (91%) | **50014 (51%)** |
| model E | 2 (0%) | 6 (0%) | 11 (0%) | 22 (0%) | 70 (1%) | **112 (0%)** | 9596 (99%) | 8499 (98%) | 7209 (97%) | 6310 (94%) | 5461 (91%) | **51368 (53%)** |
| model F | 7 (0%) | 30 (0%) | 36 (0%) | 72 (1%) | 127 (1%) | **283 (0%)** | 9505 (98%) | 8367 (97%) | 7036 (94%) | 6104 (91%) | 5202 (87%) | **50699 (52%)** |

# Table s4 additional text - Breakdown of calculations for extrapolation to UK population

Given the secular trend there are very few patients whose risk increases when comparing model A to model F. Therefore we solely focus on the proportion of patients who are initially classified as high risk, that cross the threshold to low risk. All figures in below calculations can be found in supplementary Table 6.

**1) Number of patients aged 25-84 in England (37,273,000)**

This was taken directly from the reference^1^ given = 37,273,000

**2) Proportion (number) of patients aged 25-84 eligible for risk assessment = 79% (29,382,463)**

The number of patients aged 25-84 that were registered on 1^st^ Jan 2016 and had linked data = 938,150. The number of this group that had not had a CVD event or statin treatment prior to 1^st^ Jan 2016 = 739,561. We took the ratio of these to be the proportion of patients aged 25-84 that would be eligible for risk assessment = 79%.

**3) Proportion of patients that would be classified as high risk (> 10%) = 22.64% (6,652,920)**

Of the 739,583 patients, 167,460 patients were classified as high risk = 22.64%. Therefore we assumed 22.64% of the English population aged 25-84 would be classified as high risk = 6,652,920

**4) Proportion (number) of high risk patients that would be reclassified as low risk according to model F = 57.00% (3,792,474)**

Of the 167,460 patients classified as high risk, 95,460 are reclassified to low risk = 57.00%. Therefore we assumed 57.00% of the high risk group in the English population would also be reclassified = 3,792,474.

# References

1. Statistics, O. for N. Estimates of the population for the UK, England and Wales, Scotland and Northern Ireland. (2018). at <https://www.ons.gov.uk/peoplepopulationandcommunity/populationandmigration/populationestimates/datasets/populationestimatesforukenglandandwalesscotlandandnorthernireland>
